# Effects of *Bifidobacterium bifidum* tetragonum tablets and Jin Gui Ren Qi Pill on intestinal flora and metabolism in patients with diabetic kidney disease

**DOI:** 10.3389/fphar.2024.1346168

**Published:** 2024-07-29

**Authors:** Cheng-Yu Zhang, Dong-jie Yue, Di Wang, Fei-fei Wu

**Affiliations:** ^1^ Department of Endocrinology and Metabolism, The Fifth People’s Hospital of Chongqing, Chongqing, China; ^2^ Heping Hospital Affiliated to Changzhi Medical College, Changzhi, Shanxi, China; ^3^ Zhengzhou Second People’s Hospital, Zhengzhou, Henan, China; ^4^ Department of Endocrinology and Metabolism, Binzhou People's Hospital, Binzhou, Shandong, China; ^5^ Department of Endocrinology and Metabolism, Heping Hospital Affiliated to Changzhi Medical College, Changzhi, Shanxi, China

**Keywords:** diabetic kidney disease, Bifidobacterium bifidum tetragonum tablets, Jin Gui Ren Qi Pill, intestinal flor, serum total cholesterol

## Abstract

**Objective:**

To investigate the effects of *Bifidobacterium bifidum* tetragonum tablets and Jin Gui Ren Qi Pill on intestinal flora and metabolism in patients with diabetic kidney disease.

**Methods:**

In the study conducted at Heping Hospital of Changzhi Medical College from March 2021 to December 2022, 30 cases of patients diagnosed with diabetic nephropathy were meticulously selected as study subjects. Employing a double-blind randomized table method, these patients were randomly allocated into three groups: the control group (n = 10), the *Bifidobacterium bifidum* tetragonum tablets group (n = 10), and the Jin Gui Ren Qi Pill group (n = 10). The control group received standard western medical treatments for diabetic nephropathy, including serum glucose, blood lipids, blood pressure management, and other conventional therapies. In addition to the standard treatments, the *Bifidobacterium bifidum* tetragonum tablets group received *Bifidobacterium bifidum* tetragonum tablets, while the Jin Gui Ren Qi Pill group received Jin Gui Ren Qi Pill. Before and after a 4-week treatment period, various baseline parameters were assessed, including fasting blood glucose, 2-h postprandial blood glucose, triglycerides, serum total cholesterol, serum low-density lipoprotein cholesterol, serum high-density lipoprotein cholesterol, random urine microalbumin/creatinine ratio (ACR), blood creatinine (SCr), and traditional Chinese medicine evidence scores. Stool specimens were collected from all three groups before and after treatment for 16S rDNA high-throughput sequencing, followed by comprehensive analyses including OUT clustering, Alpha diversity, Beta diversity, species composition analysis, LEfSe analysis, and KEGG function prediction. Spearman correlation analysis was employed to explore the relationship between intestinal flora and clinical indicators. Furthermore, fasting peripheral venous blood was collected from patients in the Bifidobacterium tetrapunctate tablets group and the control group before and after intervention to measure the optical density values of tumor necrosis factor-α (TNF-α), interleukin-2 (IL-2), and interleukin-6 (IL-6) using the Beijing Biolite ELISA kit. This study was conducted with the approval of the Ethics Committee of Changzhi Medical College.

**Results:**

1. The 2hPBG, total cholesterol and LDL levels were observed among patients with diabetic kidney disease (DKD) across all groups: the Jin Gui Ren Qi Pill group, the *Bifidobacterium bifidum* tetragonum tablets group, and the control group (*p* < 0.05). 2. The Jin Gui Ren Qi Pill demonstrated superior efficacy in alleviating TCM symptoms and reducing the ACR compared to both the *Bifidobacterium bifidum* tetragonum tablets group and the control group. Conversely, *Bifidobacterium bifidum* tetragonum tablets exhibited a more pronounced reduction in TC levels compared to both the Jin Gui Ren Qi Pill and control groups. Notably, *Bifidobacterium bifidum* tetragonum tablets effectively decreased (IL-2) levels in patients with DKD. 3. *Bifidobacterium bifidum* tetragonum tablets also demonstrated efficacy in reducing IL-2 levels in DKD patients. 4. Analysis of intestinal microorganism abundance and diversity before and after the intervention, as well as among the three groups, revealed no significant alterations. Similarly, comparisons of ACE, Chao, Simpson, and Shannon indices showed no statistically significant differences (*p* > 0.05). 5. Qualitative analysis of intestinal microorganisms before and after intervention, as well as among the three groups, indicated no significant differences. Anosim test results also did not reveal qualitative distinctions (Anosim test R = 0.021, *p* = 0.215). 6. LEfSe analysis unveiled a noteworthy increase in Prevotella_7 abundance within the Jin Gui Ren Qi Pill group post-intervention (*p* < 0.05). 7. Furthermore, Chinese medicine evidence scores, body mass index, TC, and LDL levels correlated positively with the relative abundance of Tyzzerella_3 bacterial flora. Conversely, age, disease duration, and 2hPBG correlated positively with the relative abundance of Christensenellaceae_R_7 flora, while TC and LDL levels displayed a negative correlation with the relative abundance of Christensenellaceae_R_7 flora.

**Conclusion:**

The combination of Jin Gui Ren Qi Pill with western medical treatment exhibited superior efficacy in ameliorating clinical symptoms and reducing the ACR in patients with DKD compared to western medical treatment alone. Furthermore, this combination therapy led to an increase in the abundance of Prevotella_7 within the intestinal flora of patients, suggesting a potential enhancement in carbohydrate metabolism by the intestinal microbiota. On the other hand, *Bifidobacterium bifidum* tetragonum tablets bacterial tablets combined with western medical treatment demonstrated enhanced efficacy in reducing TC levels in DKD patients compared to western medical treatment alone. Additionally, this combination therapy effectively reduced the levels of IL-2 in DKD patients, thus mitigating inflammation in these individuals.

## 1 Introduction

Diabetic kidney disease (DKD) stands as a prevalent and formidable microvascular complication of diabetes mellitus, affecting approximately 20%–40% of individuals with diabetes mellitus (DM), with 1/3 of DKD patients eventually progressing to end-stage renal disease (ESRD) (Ruiz-Ortega et al., 2020; Sun et al., 2022). Presently, scholarly consensus points towards altered renal hemodynamics, dysregulation of the renin-angiotensin-aldosterone system (RAS), oxidative stress, inflammatory responses, and fibrosis as the primary players in DKD pathogenesis (Tan et al., 2019). Symptomatic treatment regimens, including intensive glycemic control, cholesterol level management, and renin-angiotensin system blockade for blood pressure regulation, are commonly administered in clinical settings to slow the progression of diabetic kidney disease (DKD). However, these approaches have been shown to be insufficient in preventing the advancement of DKD to end-stage renal disease (ESRD) (Arora and Singh, 2013; Alicic et al., 2017). Hence, the quest for novel therapeutic strategies and a deeper understanding of DKD mechanisms persists, holding profound implications for prolonging survival and enhancing the quality of life for DKD patients.

The gut microbiota (GM) actively contributes to host immunity and sustains internal environment homeostasis by participating in functions such as scavenging and defending against pathogenic bacteria (Krishnan et al., 2015). Certain probiotic genera in the gut microbiota enhance host resistance to infectious diseases and produce beneficial metabolites, such as short-chain fatty acids (SCFAs), which are known to reduce inflammation (Zaky et al., 2021). SCFAs serve as a major energy source for enterocytes and colonocytes, and inhibit intestinal inflammation and oxidative stress (Aoki et al., 2017; Oliveira et al., 2019). Furthermore, SCFAs exert anti-inflammatory effects by inhibiting protein deacetylases, thereby controlling the release of pro-inflammatory cytokines (IL-6, TNF-α, IL-18) (Tan et al., 2014). In addition, certain genera release lipopolysaccharides (LPS) and other pro-inflammatory substances that possess strong immune activation capabilities (Cani et al., 2008). These can induce the release of pro-inflammatory cytokines (TNF-α, IL-2, IL-6, etc.), triggering an inflammatory cascade that exacerbates kidney damage (Salguero et al., 2019; Wang and Yang, 2019). Therefore, regulating the abundance of beneficial bacteria in the gut microbiota and reducing the production of harmful substances are crucial for controlling the progression of diabetic nephropathy.

Microecological preparations encompass formulations derived from probiotics, their metabolites, and growth-promoting substances, chiefly comprising various probiotics and prebiotics. These preparations are tailored to ameliorate dysbiosis within the body (Leustean et al., 2018). By augmenting the content of beneficial bacteria, microecological preparations have the capacity to rectify intestinal microbiota disorders, diminish intestinal permeability, and curtail endotoxin production. Consequently, they play a pivotal role in attenuating intestinal inflammatory responses and slowing the progression of chronic kidney disease (CKD) (Jia et al., 2018; Zhu, 2018). Several studies, both domestic and international, have demonstrated that certain beneficial bacteria can effectively improve the intestinal flora environment, enhance renal function, and alleviate inflammatory responses in patients with diabetes mellitus and diabetic kidney disease (DKD) (Ni et al., 2013; Mazruei Arani et al., 2018; Su and Chen, 2020; Guoliang et al., 2021; Tan and Zhou, 2021). Bifidobacterium tetragonum tablets, commonly used microecological agents, contain four beneficial bacteria (*Bifidobacterium bifidum*, *Lactobacillus* acidophilus, *Enterococcus faecalis*, *Bacillus* cereus) that are effective in correcting disorders of glucose and lipid metabolism in patients. However, research on their therapeutic effects in diabetic nephropathy remains insufficient. This study aims to explore whether *Bifidobacterium bifidum* tetragonum tablets, a hybrid microecological preparation, can rapidly colonize the gastrointestinal tract of DKD patients to establish a new and stable intestinal microecological balance. Additionally, it investigates whether short-term intervention with exogenous probiotic bacteria can successfully reduce the production of inflammatory factors in DKD patients, thereby improving renal function and delaying DKD progression. This aspect has not been addressed in previous studies.

In the realm of traditional Chinese medicine (TCM), the treatment of DKD holds promise in effectively alleviating clinical symptoms and retarding disease progression. Both clinical observations and animal experiments have revealed that numerous TCM remedies possess the capacity to lower blood glucose levels and forestall renal function decline by rectifying GM dysregulation in DKD patients or DKD mouse models (Dai et al., 2017; Qi et al., 2020). Rooted in Chinese medicine theory, Jin Gui Ren Qi Pill has been deployed in diabetes mellitus treatment since the Eastern Han Dynasty era. Comprising dihuang, yam, cornelian cherry, ze diarrhea, poria, mudanpi, gui zhi, and epimedium, Jin Gui Ren Qi Pill represents an ancestral kidney-tonifying formula (Tian et al., 2020), ideally suited for managing diabetic nephropathy. In clinical investigations, Jin Gui Ren Qi Pill exhibited notable efficacy in ameliorating blood glucose levels, blood lipids, blood pressure, and renal function among patients with DKD. Studies (Chen et al., 2019) have shown that Jin Gui Ren Qi Pill treatment significantly reduces fasting blood glucose (FBG), 24-hour urine protein quantification, serum creatinine (SCr), and blood urea nitrogen (BUN) levels in patients with diabetic kidney disease (DKD). Experimental studies indicate that Jin Gui Ren Qi Pill has multiple beneficial effects, including improving insulin resistance, reducing inflammatory states, mitigating oxidative stress, protecting renal podocytes, and delaying renal fibrosis (Huang and Xiang, 2019; Wang et al., 2021). However, its specific mechanism of action in treating diabetic nephropathy remains inadequately researched. We hypothesize that Jin Gui Ren Qi Pill may improve renal function by modulating the intestinal bacterial flora. Additionally, we aim to compare the effects of this Chinese herbal preparation with microecological preparations on the improvement of clinical symptoms and laboratory indicators in DKD patients, an area not previously addressed in studies.

## 2 Materials and methods

### 2.1 Research object

Thirty patients diagnosed with diabetic nephropathy, treated at Heping Hospital affiliated with Changzhi Medical College between March 2021 and December 2022, were carefully chosen for this study. Each patient met the diagnostic criteria outlined in Western medical guidelines for diabetic nephropathy, as well as the Chinese medicine evidence criteria validating the use of Jin Gui Ren Qi Wan. Employing the randomized numerical table method, the selected patients were meticulously divided into three groups: the Jin Gui Ren Qi Pill group (10 patients), the *Bifidobacterium bifidum* tablet group (10 patients), and the control group (10 patients).

#### 2.1.1 Diagnostic criteria

In this study, the diagnostic criteria for diabetic nephropathy were delineated based on the Western medical guidelines derived from the Chinese Guidelines for the Diagnosis and Treatment of Diabetic Kidney Disease (Expert Group of Chinese Society of Nephrology, 2021) and the Chinese Guidelines for the Prevention and Control of Type 2 Diabetes Mellitus (2020 Edition) (Diabetes Society, 2021). The established criteria are as follows: 1. Confirmation of diabetes mellitus history, characterized by typical symptoms such as polyuria, polydipsia, polyphagia, and emaciation, accompanied by a fasting blood glucose (FBG) level ≥7.0 mmol/L or random serum glucose (Glu) level ≥11.0 mmol/L or oral glucose tolerance test (OGTT) 2-hour postprandial glucose level (2 h) ≥11.0 mmol/L, or glycated hemoglobin (HbA1c) level ≥6.5%. 2. Diagnosis of diabetic kidney disease (DKD) may be established upon the presence of a causal relationship with urinary protein and alterations in renal function, provided that other primary and secondary glomerular and renal systemic disorders have been ruled out. DKD diagnosis can be confirmed if one of the following conditions is met: a. Random urine albumin/creatinine ratio (UACR) ≥30 mg/g, with repeat testing demonstrating UACR ≥30 mg/g within 3–6 months. b. Estimated glomerular filtration rate (eGFR) persistently <60 mL/min-1.73 m^2^ for more than 3 months. c. Histopathological findings from renal biopsy consistent with the characteristic pathological changes of DKD.

The diagnostic criteria for Traditional Chinese Medicine (TCM) patterns were established by consulting the Guiding Principles for Clinical Research of New Chinese Medicines (for trial implementation) (Xiaoyu, 2002). Primary symptoms encompass manifestations such as depressed spirit, cold limbs, diarrhea, impotence, and spermatorrhea, while secondary symptoms include features like a pale and dull complexion, tiredness and weakness, swelling of the face and eyes, as well as lumbago and tinnitus. Tongue examination typically reveals a pale tongue covered with white moss, and pulse palpation reveals a slow or weak pulse. To confirm the diagnosis of the aforementioned symptoms, patients must present with either 2 of the primary symptoms or 1 primary symptom combined with 1 secondary symptom.

#### 2.1.2 Inclusion criteria

1. Diagnosed with diabetic nephropathy in accordance with the above diagnostic criteria for diabetic nephropathy in Western medicine; and also in accordance with the above diagnostic criteria for Chinese medicine. 2. Aged 30–80 years old; 3. With a disease duration of >6–10 years (Andrade-Oliveira et al., 2015); 4. Patients with diabetic nephropathy in the Morgensen staging phase III-IV (Diabetes Society, 2021); 5. Signing a self-administered informed consent form to participate voluntarily; and 6. Outpatients or hospitalized patients with a long term follow-up period. Or hospitalized patients.

#### 2.1.3 Exclusion criteria

1. Combination of primary and other secondary renal diseases, such as diagnosed glomerulonephritis, IgA nephropathy, nephrotic syndrome, hypertensive nephropathy, etc.; 2. Acute complications of diabetes mellitus, such as diabetic ketoacidosis, hyperglycemic and hyperosmolar state, or infections, stress; 3. History of myocardial infarction or stroke, or the presence of serious cardiovascular disease and risk within 6 months; liver function abnormalities; other endocrine diseases (hyperthyroidism, Cushing’s syndrome), rheumatic system diseases, hematologic diseases, malignancies; 4. Patients with coexisting urinary tract infections, end-stage renal failure, patients who have received or are currently receiving dialysis treatment; 5. Participation in other research projects within the last 3 months or two or more projects within 1 year; 6. Serious contraindications to the study medication, pregnant women; 7. Severe mental disorders; 8. Use of medications that may affect the evaluation of clinical outcomes, such as antibiotics within the last 2 weeks; and oral intake within the last 3 months or currently of Probiotic drugs that regulate intestinal flora.

### 2.2 Research methods

#### 2.2.1 Grouping

Thirty DKD patients who met the inclusion criteria were divided into three groups according to the randomized double-blind method 1:1:1: 1. Control group: basal drug therapy (insulin or oral hypoglycemic drugs, antihypertensive drugs, lipid regulating drugs) for 4 weeks; 2. Jin Gui Ren Qi Pill group: basal drug therapy (insulin or oral drugs for lowering glucose, lowering blood pressure, and regulating lipids) + Jin Gui Ren Qi Pill (5 g/dose, 2 times/dose, Beijing Tong Ren Tang) for 4 weeks 3. *Bifidobacterium bifidum* tetragonum tablets group: basic drug therapy (insulin or oral drugs for lowering glucose, lowering blood pressure, and regulating lipids) + *Bifidobacterium bifidum* tetragonum tablets (1.5 g/times, 3 times/day, Hangzhou Yuanda Bio-Pharmaceutical Co., Ltd.) for 4 weeks.

Meanwhile all three groups of DKD patients received lifestyle interventions and diabetes health education as part of the standard treatment. Participants were asked not to change their usual diet and exercise levels during the trial. The trialists followed up with the participants weekly to ask about any symptoms or side effects of the medication. Participants will be excluded from the trial if they do not take their medication dose or exceed 10% of the dose given. All participants will be asked to review the results at week 5.

#### 2.2.2 Research steps and processes

Before initiating treatment, comprehensive patient information was gathered, including clinical symptoms evaluated via the TCM evidence score. Additionally, parameters such as FBG, 2hPBG, TG, TC, LDL-C, HDL-C, SCr, ACR. Fecal specimens were collected and stored at −80°C, and 3 mL of fasting elbow vein blood was drawn in the early morning to measure the optical density (OD) values of TNF-α, IL-2, and IL-6. These indices were reassessed after 4 weeks of treatment to gauge changes in the TCM evidence score.

Subsequently, a comparison was conducted on the general characteristics and clinical indicators among the three groups of subjects. Microbial diversity testing was performed on fecal specimens to delineate differences in microbial composition pre- and post-medication administration across the three groups. Moreover, disparities in serum inflammatory factor levels (IL-2, IL-6, and TNF-α) between the *Bifidobacterium bifidum* tetragonum tablets group and the control group were analyzed before and after medication administration. The improvement in clinical symptoms, as reflected by the TCM evidence score, was compared among the three groups of subjects.

##### 2.2.2.1 General data collection

The study subjects’ particulars, including name, gender, age, height, weight, blood pressure, history of DM, current therapeutic medications, and calculation of body mass index (BMI), were meticulously documented. BMI was calculated using the formula: BMI = weight/height (kg/m^2^).

##### 2.2.2.2 Evaluation of clinical manifestations

The clinical presentations of both patient groups before and after medication administration were evaluated using the Traditional Chinese Medicine evidence scale (Table 1). This scale was developed in accordance with the guidelines outlined in the Guiding Principles for Clinical Research of New Chinese Medicines (Trial Implementation) (Xiaoyu, 2002).

**TABLE 1:** Traditional Chinese medicine evidence scale table of DKD.

Item		Standard for evaluation				Score
		0	2	4	6	
Primary symptom	Be languid	No	Often tired It does not affect life	Low spirit, forgetfulness and drowsiness, affect the life	Weak spirit, unclear words	
	Cold limbs	No	Micro-feel cold or only cold hands and feet	Hand and foot reverse cold, Thick clothes	Fear of cold	
	Diarrhea	No	The stool is not formed 1–2 Times a day	The stool is thin Three to four times a day	The stool is thin More than 5 times a day	
	Impotence	No	The sex drive is OK Not long after	Libido weakened Forced erection	Libido disappears No erection	
	Spermatorrhea	No	Spermatogenesis occurs every 2–3 days	Spermatoses more than 2 times a night, but no spermatoses	spermatorrhea	
		0		1		
Secondary disease	The face is pale	No		Yes		
	Exhaustion and fatigue	No		Yes		
	Edema of the face	No		Yes		
	Lumbago and tinnitus	No		Yes		
Aspect of the tongue		Normal		Pale tongue color, white and greasy tongue coating		
Pulse condition		Normal		Pulse sinking late or thin weak		

##### 2.2.2.3 Blood, urine and stool samples were collected and tested

1. Blood and urine specimen collection and testing: Upon admission and after 4 weeks of treatment, blood specimens were collected from all study subjects following an overnight fast. Blood was drawn from the elbow vein in the early morning, and urine specimens were also collected. The following parameters were assessed: FBG, 2hPBG, TC, TG, LDL, HDL, ACR, SCr.
2. Fecal specimen collection and testing: Fecal specimens were obtained from all study subjects upon admission and after 4 weeks of treatment. These specimens were stored in an ultra-low-temperature refrigerator at −80°C. Subsequently, they were sent to Shanghai Baoteng Biomedical Technology Co., Ltd. for fecal flora extraction and quality control, sample amplification, and 16S rDNA high-throughput sequencing. Advanced bioinformatic analysis and statistical comparisons were conducted.
3. Inflammatory factor detection: Fasting elbow venous blood samples (3 mL) were collected in the early morning and anticoagulated with heparin. After standing for 30 min, the samples were centrifuged at 3,000 rpm for 30 min to obtain the supernatant. Sandwich ELISA was performed using Biolite’s ELISA kits for the detection of IL-2, IL-6, and TNF-α, along with the enzyme-labeled immunoassay detector.

### 2.3 Data statistics and analysis

The statistical analysis was conducted using SPSS 26.0 software, wherein all data underwent normality testing and chi-square tests before analysis. For normally distributed data, mean ± standard deviation ( $\bar{x}$ ± s) was utilized, while non-normally distributed data were represented by median (upper and lower quartiles) *M (P25, P75)*. Count information was presented as frequency and rate (%). For comparisons between groups of two sample means, paired-samples t-tests (for normal distribution) or Mann-Whitney U tests (for non-normal distribution) were employed. Comparisons involving more than two sample means utilized one-way analysis of variance, supplemented by Kruskal-Wallis H test in cases of variance heterogeneity. Comparisons of count data between groups were performed using chi-square tests (χ^2^) or Fisher’s test. QIIME software and R package were utilized to calculate α and β diversity, facilitating the comparison of intestinal flora differences among samples. Spearman correlation analysis was employed for bivariate correlation assessments. The significance level was set at α = 0.05, and a *p* < 0.05 was considered indicative of a statistically significant difference.

## 3 Fruit

### 3.1 General data comparison

This study included a total of 30 patients, with 10 cases in the Jin Gui Ren Qi Pill group, 10 cases in the *Bifidobacterium bifidum* tetragonum tablets group, and 10 cases in the control group. The three patient groups in this study demonstrated good compliance, adhering to the prescribed medication regimen as verified through investigator follow-up. In the *Bifidobacterium bifidum* tetragonum tablets group, there was one case of diarrhea, which resolved after 3 days of conventional treatment.

There were no statistically significant differences observed in the male-to-female ratio, age distribution, medical history, systolic and diastolic blood pressure, and BMI among the three groups (*p* > 0.05). Furthermore, there was no significant variance noted in the utilization of hypoglycemic medications across the three groups (*p* > 0.05). As shown in Table 2.

**TABLE 2:** Comparison of general data of the three groups [( $\bar{x}$ ± s) or (%)].

Clinical index	G1 (N = 10)	G2 (N = 10)	G3 (N = 10)	Statistics	*P*-valve
Sex (male)	3 (30%)	6 (60%)	6 (60%)	χ^2^ = 2.400	0.301
Age	60.40 ± 8.579	62.6 ± 10.967	60.60 ± 11.167	*F* = 0.139	0.871
Duration of diabetes (year)	15.60 ± 6.275	13.1 ± 4.977	13.50 ± 7.261	*F* = 0.463	0.634
Systolic pressure (mmHg)	129.30 ± 11.167	143.3 ± 28.860	144.30 ± 19.505	*F* = 1.210	0.314
Diastolic pressure (mmHg)	78.80 ± 13.423	79.70 ± 12.789	87.7 ± 10.111	*F* = 1.615	0.218
BMI(kg/m^2^)	22.948 ± 4.907	23.117 ± 3.426	25.783 ± 1.774	*F* = 1.947	0.162
Hypoglycemic agent n (%)				χ^2^ = 3.525	0.474
Insulin	2 (20%)	1 (10%)	1 (10%)		
Insulin + metformin	3 (30%)	7 (70%)	6 (60%)		
Insulin + acarbose	5 (50%)	2 (20%)	3 (30%)		
Take hypotensor n (%)				χ^2^ = 2.224	0.329
Yes	5 (50%)	8 (80%)	6 (60%)		
No	5 (50%)	2 (20%)	4 (40%)		

G1: Jin gui ren qi pill group, G2: *Bifidobacterium bifidum* tetragonum tablets group, G3: Control group, *p* < 0.05, there was statistical significance.

### 3.2 Clinical indicators before intervention

There was no significant difference in the values of 2hPBG, HbA1c, TC, TG, LDL, HDL, ACR, TG, SCr, eGFR, and Chinese medicine evidence scores of the three groups of patients before the intervention (*p* > 0.05); patients in the group of *Bifidobacterium bifidum* tetragonum tablets had low FBG compared with the control group, and the difference was statistically significant (*p* = 0.026). See Table 3.

**TABLE 3:** Pre-intervention clinical indicators of the three groups.

Clinical index	G1 (N = 10)	G2 (N = 10)	G3 (N = 10)	Statistics	*P*-valve
FBG (mmol/L)	10.203 ± 3.497	7.586 ± 4.522	11.928 ± 4.289^a^	*F* = 2.808	0.078
2hPBG (mmol/L)	21.831 ± 4.088	16.825 ± 7.512	19.87 ± 4.029	*F* = 2.136	0.138
HbA1c (%)	9.07 ± 2.368	8.570 ± 2.020	9.70 ± 1.975	*F* = 0.708	0.502
TC (mmol/L)	3.911 ± 0.892	4.087 ± 1.013	4.51 ± 1.316	*F* = 0.806	0.457
LDL (mmol/L)	2.348 ± 0.642	2.397 ± 0.857	2.911 ± 0.969	*F* = 1.399	0.264
HDL (mmol/L)	1.034 ± 0.218	1.185 ± 0.487	1.034 ± 0.197	*F* = 0.706	0.503
eGFR (mL/min/1.73 m^2^)	95.766 ± 35.720	91.257 ± 42.148	108.426 ± 27.437	*F* = 0.625	0.543
TG (mmol/L)	1.395 (1.135, 2.50)	1.225 (0.973, 1.562)	1.340 (1.015, 2.61)	*H =* 2.073	0.355
ACR (ug/umoL)	182.35 (102.583, 668.078)	229.185 (58.175, 2396.578)	228.945 (59.168, 696.795)	*H =* 0.163	0.922
SCr (umol/L)	68.00 (47.00, 91.25)	73.00 (63.75, 90.75)	60.00 (56.50, 68.75)	*H =* 2.784	0.249
TCM syndrome score	5.300 ± 1.703	5.102 ± 0.214	4.900 ± 0.994	*F* = 0.842	0.365

G1: Jin Gui Ren Qi Pill group, G2: *Bifidobacterium bifidum* tetragonum tablets group, G3: Control group.

^a^ *P*: G2 to G3 ratio *p* < 0.05, *p* < 0.05 had statistical significance.

### 3.3 Clinical indicators after intervention

After intervention, there were no significant differences in systolic blood pressure, diastolic blood pressure, FGB, TC, TG, LDL, HDL, ACR, SCr, and eGFR equivalence among three groups (*p* > 0.05). After intervention, 2hPBG was significantly higher in *Bifidobacterium bifidum* tetragonum tablets group than in the other two groups, while the median TCM syndrome score in Jin Gui Ren Qi Pill group was lower, with statistical significance (*p* < 0.001). See Table 4.

**TABLE 4:** Clinical indicators of the three groups after intervention.

Clinical index	G1 (N = 10)	G2 (N = 10)	G3 (N = 10)	Statistics	*P*-valve
Systolic pressure (mmHg)	122.80 ± 10.56	130.8 ± 17.061	136.30 ± 17.852	*F* = 1.917	0.167
Diastolic pressure (mmHg)	73.2 ± 9.09	75.3 ± 10.51	80.80 ± 2.407	*F* = 1.841	0.178
FBG (mmol/L)	5.77 ± 1.062	6.209 ± 1.655	6.24 ± 0.68	*F* = 0.479	0.625
2hPBG (mmol/L)	7.76 ± 1.152^a^	10.609 ± 2.029^b^	7.83 ± 1.065	*F* = 12.043	**0.000**
TC (mmol/L)	3.63 ± 0.267	3.448 ± 0.989	4.397 ± 1.282	*F* = 2.190	0.131
LDL (mmol/L)	2.172 ± 0.609	2.003 ± 0.724^b^	2.841 ± 0.932	*F* = 3.341	0.051
HDL (mmol/L)	1.12 ± 0.238	1.031 ± 0.282	1.066 ± 0.176	*F* = 0.361	0.700
eGFR (mL/min/1.73 m2)	97.353 ± 28.535	85.557 ± 34.174	106.14 ± 21.374	*F* = 1.312	0.286
TG (mmol/L)	1.35 (1.118, 2.288)	0.745 (0.628, 1.578)	1.280 (1.002, 2.42)	*H* = 4.985	0.082
ACR (ug/umol)	101.275 (48.123, 352.073)	343.74 (87.825, 1782.428)	181.11 (52.223, 541.478)	*H* = 2.418	0.298
SCr (umol/L)	64.50 (50.00, 75.00)	71.50 (68.50, 87.25)	62.00 (55.25, 68.50)	*H* = 5.919	0.052
TCM syndrome score	1.7 ± 0.675	2.9 ± 0.478	3.2 ± 0.632	*F* = 2.979	**0.000**

G1: Jin Gui Ren Qi Pill group, G2: *Bifidobacterium bifidum* tetragonum tablets group, G3: Control group.

Bold text in the article represents the prompt *p* < 0.05.

^a^ *P*: G1 and G2 ratio *p* < 0.05.

^b^ *P*: G2 and G3 ratio *p* < 0.05, *p* < 0.05 was statistically significant.

### 3.4 Analysis of inflammatory factors before and after intervention

There was no significant difference in the values of IL-2, IL-6, and TNF-α in patients in the *Bifidobacterium bifidum* tetragonum tablets group compared to the control group before intervention (*p* > 0.05). There was a significant correlation between the decrease in IL-2 values of patients in the *Bifidobacterium bifidum* tetragonum tablets group after intervention compared to pre-intervention (*p* = 0.041), and there was no significant difference in the values of IL-6 and TNF-α before and after intervention (*p* > 0.05). There was no significant difference in the values of IL-2, IL-6, and TNF-α in patients in the control group after intervention compared to before intervention (*p* > 0.05). See Table 5.

**TABLE 5:** Comparison of inflammatory factors before and after intervention between *Bifidobacterium bifidum* tetragonum tablets tablet group and control group.

OD value of inflammation index	Bifidobacterium quadruple live bacteria tablet group pre-intervention (N = 10)	Bifidobacterium quadruple live bacteria tablet group post-intervention (N = 10)	Control group pre-intervention (N = 10)	Control group post-intervention (N = 10)
IL-2 (pg/mL)	1,440.43 (1,366.94, 1,566.95)	1,254.82* (1,181.68, 1411.17)	1,367.72 (1,186.40, 1171.65)	1,461.34 (1,164.30, 1541.13)
IL-6 (pg/mL)	1,005.25 ± 299.87	989.69 ± 446.12	1,192.31 ± 369.73	1,255.99 ± 458.8
TNF-α (pg/mL)	1,283.06 ± 345.37	1,285.58 ± 376.12	1,574.58 ± 469.34	1,692.55 ± 551.48

The results of *Bifidobacterium bifidum* tetragonum tablets group were compared after intervention and before intervention, *p < 0.05. *p* < 0.05 was statistically significant.

### 3.5 Analysis of intestinal flora diversity

#### 3.5.1 OTU cluster analysis

**FIGURE 1:**
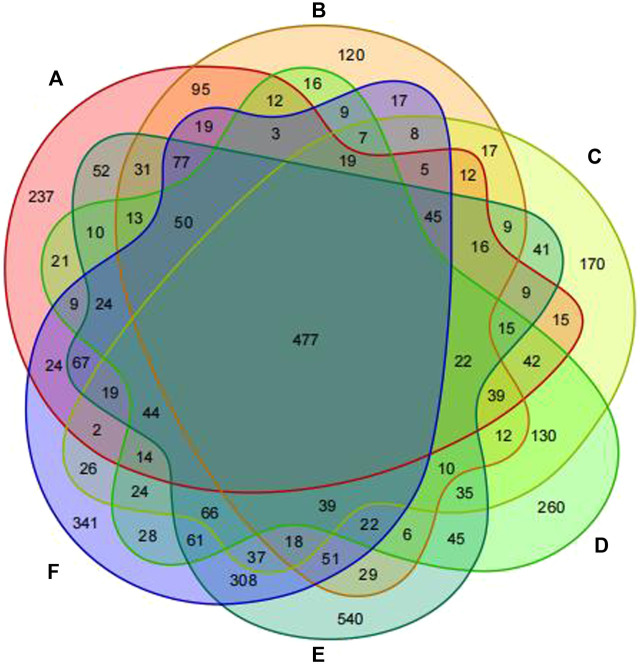
Venn diagram of six groups of samples at the OTU clustering level. Note: Group A: pre-intervention with Jin Gui Ren Qi Pill, Group B: post-intervention with Jin Gui Ren Qi Pill, Group C: control group pre-intervention, Group D: control group post-intervention, Group E: pre-intervention with *Bifidobacterium bifidum* tetragonum tablets, Group F: post-intervention with *Bifidobacterium bifidum* tetragonum tablets.

Based on the results of 16S rDNA high-throughput sequencing of fecal samples, OTU cluster analysis was conducted at a 97% similarity level, with each OTU representing a set of similar sequences. Utilizing the outcomes of the OTU cluster analysis, a Venn diagram was constructed to illustrate the overlapping area of elements among six groups: Group A (pre-intervention with Jin Gui Ren Qi Pill), Group B (post-intervention with Jin Gui Ren Qi Pill), Group C (control group pre-intervention), Group D (control group post-intervention), Group E (pre-intervention with *Bifidobacterium bifidum* tetragonum tablets), and Group F (post-intervention with *Bifidobacterium bifidum* tetragonum tablets). This visualization facilitated the comparison of the number of common and unique OTUs across different groups. The Venn diagram revealed 477 common OTU overlaps among the six groups, as depicted in Figure 1.

#### 3.5.2 Analysis of Alpha diversity index

**FIGURE 2:**
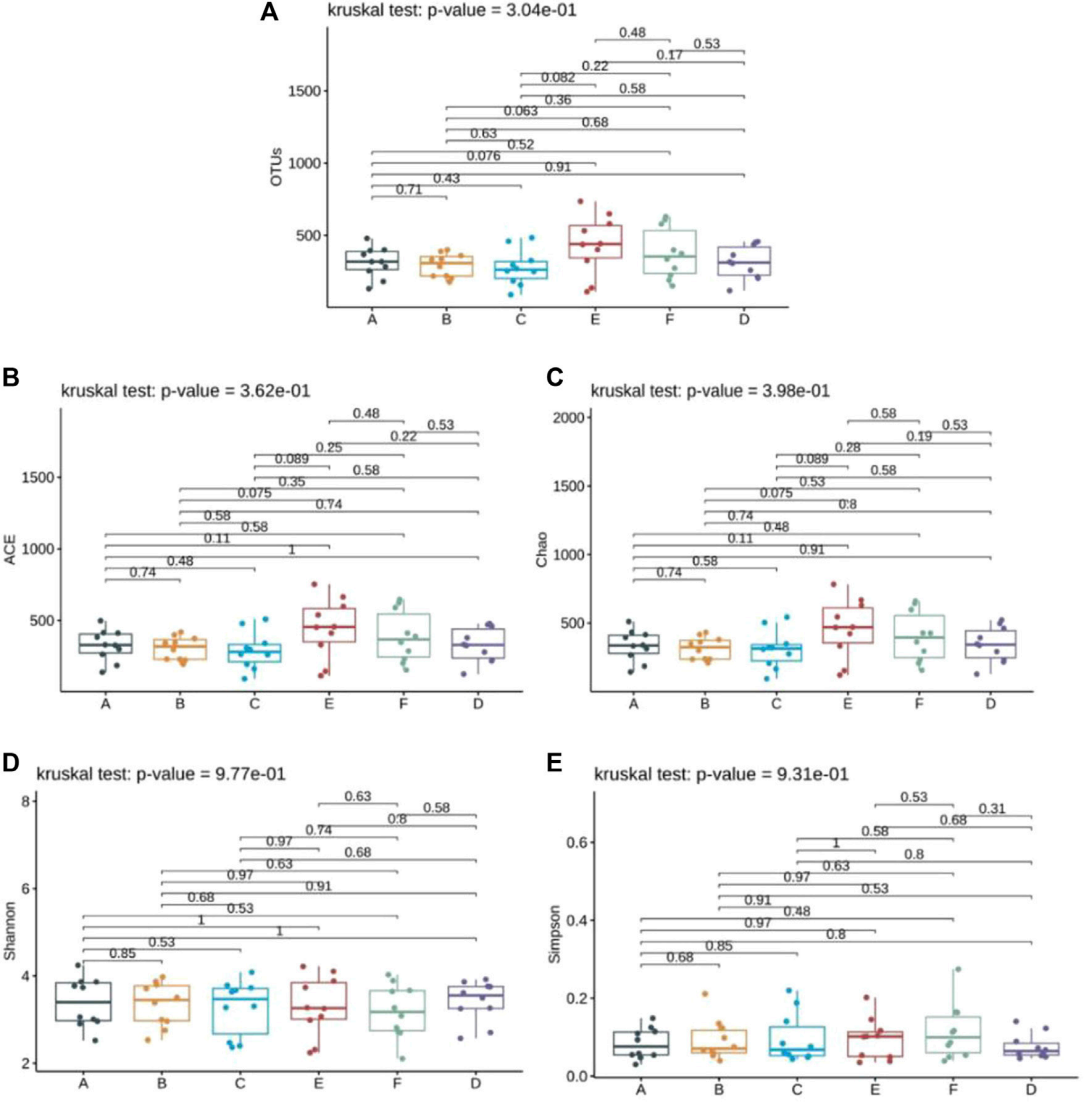
Analysis of Alpha diversity index of six groups of samples. Note: Group A: pre-intervention with Jin Gui Ren Qi Pill, Group B: post-intervention with Jin Gui Ren Qi Pill, Group C: control group pre-intervention, Group D: control group post-intervention, Group E: pre-intervention with *Bifidobacterium bifidum* tetragonum tablets, Group F: post-intervention with *Bifidobacterium bifidum* tetragonum tablets.

Alpha diversity indices serve as valuable tools for assessing both the richness and diversity of fungal species within a single sample. The ACE and Chao indices estimate the actual number of species present in the community, reflecting community richness, while the Shannon and Simpson indices gauge community diversity. Additionally, OTU indices provide a straightforward count of OTUs within samples. The findings revealed no significant disparity in the OTU counts among the six specimen groups (*p* > 0.05), suggesting similarity in the species genus composition across all groups (Figure 2A). The ACE and Chao indices further underscored this similarity, indicating no noteworthy difference in community richness among patients receiving Jin Gui Ren Qi Pill, *Bifidobacterium bifidum* tetragonum tablets, and the control group, both before and after intervention (*p* > 0.05) (Figures 2B, C). Similarly, the Shannon and Simpson indices revealed no statistically significant discrepancy in intestinal flora diversity among patients in the Jin Gui Ren Qi Pill group, the *Bifidobacterium bifidum* tetragonum tablets group, and the control group, before and after intervention (*p* > 0.05) (Figures 2D, E).

### 3.6 Analysis of microbial composition and differences between groups

#### 3.6.1 Analysis of microbial flora composition at each classification level

**FIGURE 3:**
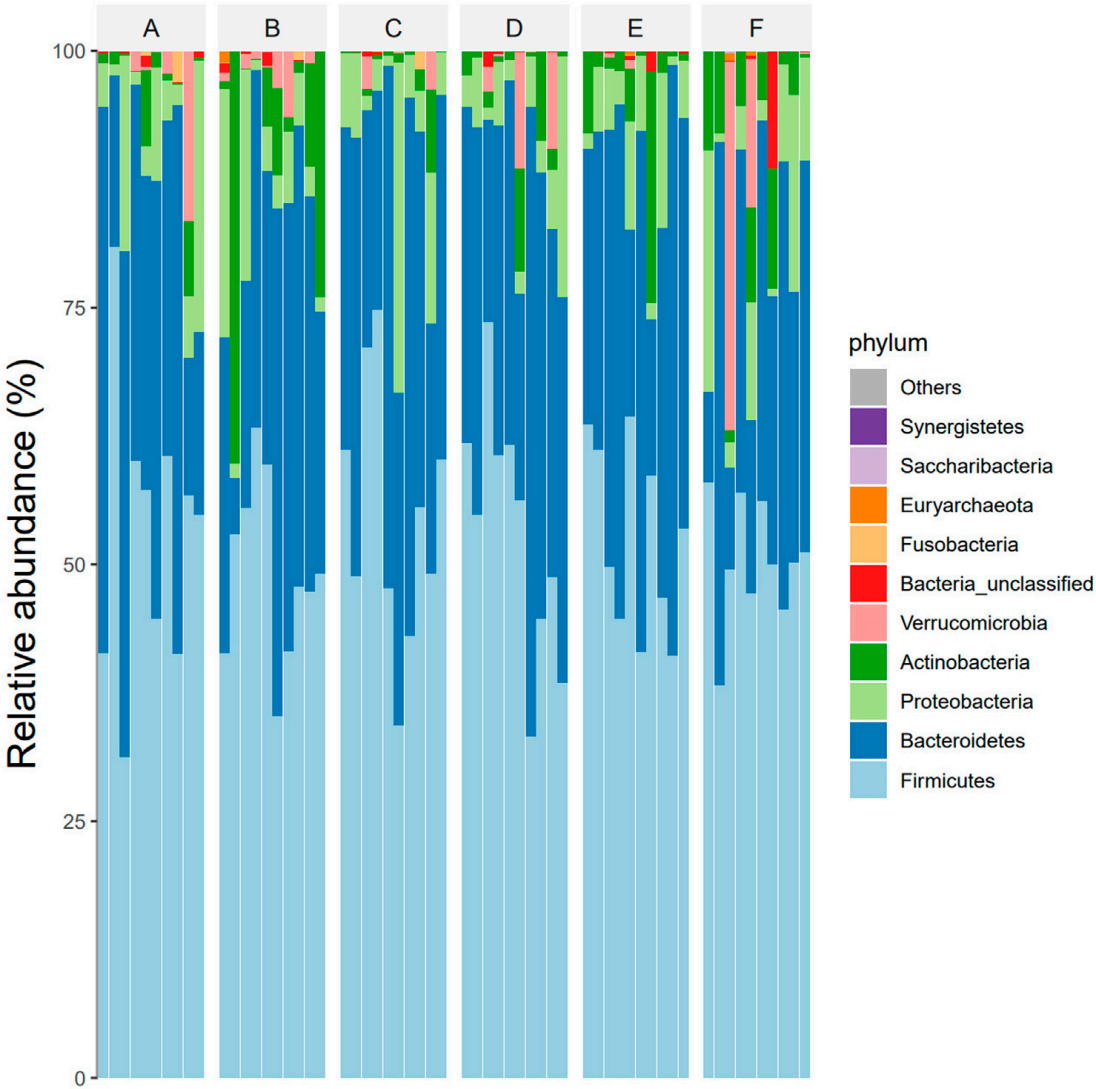
Species composition abundances of six groups of samples at phylum level. Note: Group A: pre-intervention with Jin Gui Ren Qi Pill, Group B: post-intervention with Jin Gui Ren Qi Pill, Group C: control group pre-intervention, Group D: control group post-intervention, Group E: pre-intervention with Bifidobacterium bifidum tetragonum tablets, Group F: post-intervention with Bifidobacterium bifidum tetragonum tablets.

According to the OTU clustering and species annotation results, the top 10 species common to each group at different classification levels (phyla, class, order, family, genus) were selected and the others were classified as other to draw the relative abundance histogram, and the proportion of flora composition among each group could be obtained. At the phylum level, the dominant bacteria in the intestinal tracts of the six groups were Firmicutes, Bacteroidetes, Proteobacteria, Actinobacteria and Verrucomicrobia. The abundance ratios of Firmicutes in groups A, B, C, D, E, and F were 51.9%, 48.5%, 54.3%, 53.2%, 51.2%, 49.1%, and Bacteroidetes were 35.9%, 33.5%, 35.6%, 35.9%, 37.1%, 29.8%, respectively. The abundance ratios of Proteobacteria were 7.6%, 7.1%, 8.0%, 5.7%, 7.5%, and 6.7%, and the abundance ratios of *actinomyces* were 2.0%, 9.1%, 1.3%, 2.6%, 3.0%, and 8.6%, respectively. The abundance ratios of verrucomicrophylla were 2.1%, 1.4%, 0.7%, 2.3%, 0.2%, 3.1%, respectively (see Figure 3).

**FIGURE 4:**
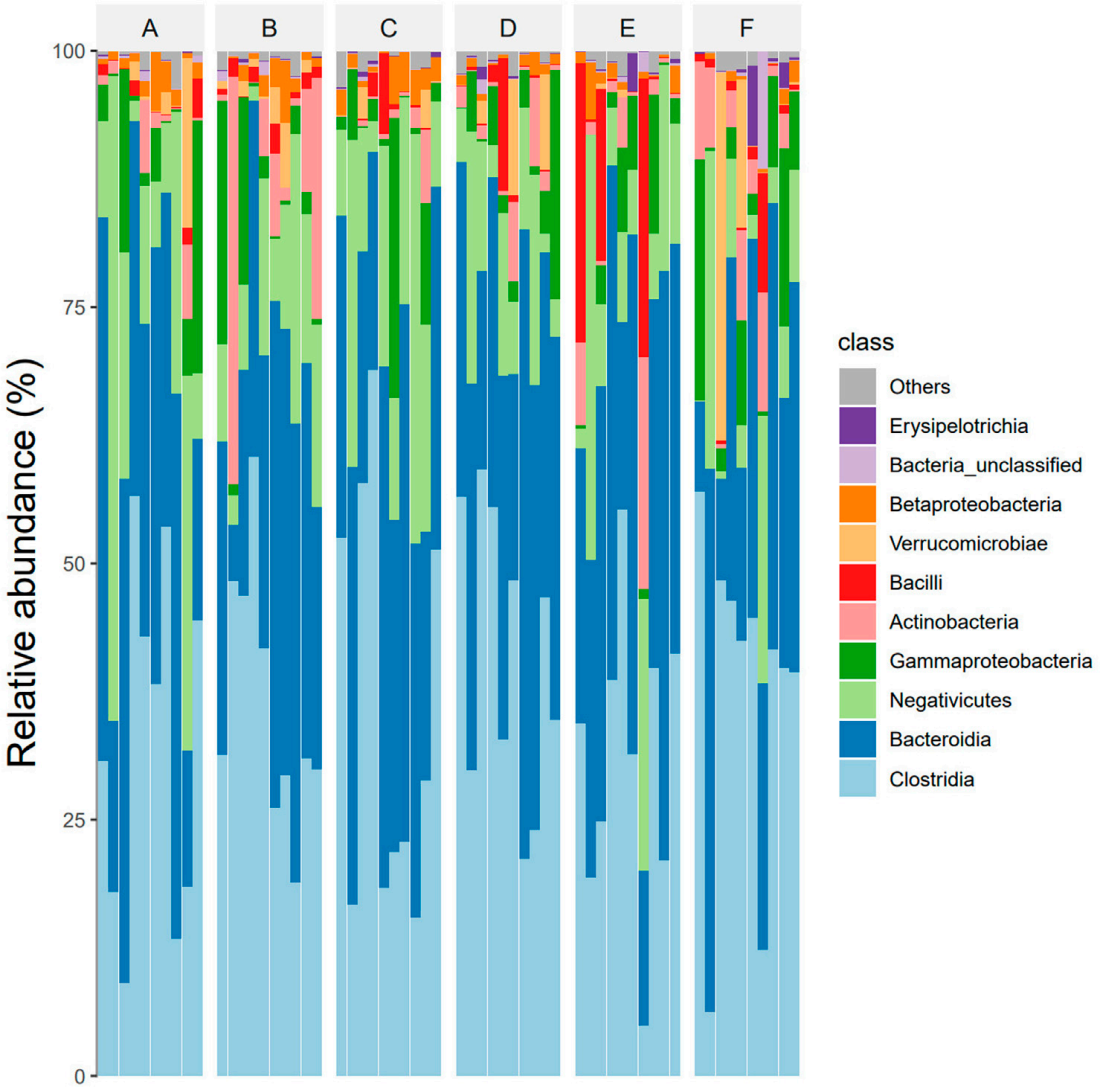
Species composition abundances of six groups of samples at class level (Note: Ibid).

At the class level, The dominant intestinal bacteria in the six groups were Clostridia, Bacteroidia, Negativicutes, Gammaproteo bacteria and Actinobacteria. The abundance ratios of *Clostridium* in groups A, B, C, D, E, and F were 45.2%, 41.7%, 47.0%, 45.7%, 40.3%, and 41.9%, and the abundance ratios of *Bacteroides* were 26.9%, 24.5%, 25.6%, 25.9%, 26.1%, and 24.7%, respectively. The abundance ratios of Firmicutes were 19.1%, 16.3%, 18.9%, 17.4%, 15.9%, 15.2%, respectively. The abundance ratios of Proteobacteria were 5.9%, 5.2%, 5.3%, 4.6%, 3.9%, 5.3%, respectively. The abundance ratios of actinomycetes were 1.9%, 8.9%, 1.2%, 2.3%, 3.1%, 5.3%, respectively (see Figure 4).

**FIGURE 5:**
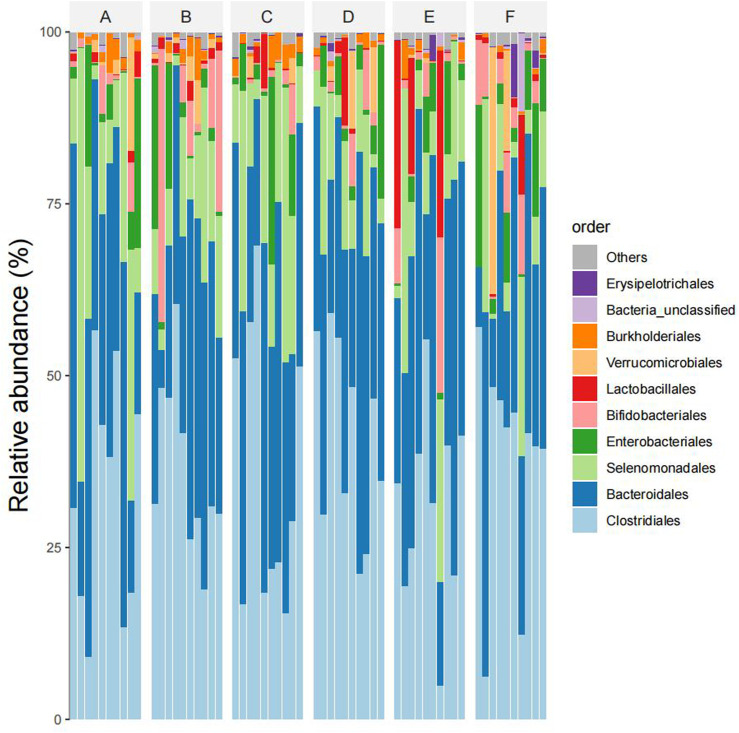
Species composition abundency map of six groups of samples at the order level (Note: Ibid).

At eye level, The dominant intestinal bacteria in the six groups were Clostridiales, Bacteroidiales, Selenomonadales, Enterbacteriales and Bifidobacterial es). The abundance ratios of Clostridiales in groups A, B, C, D, E, and F were 45.2%, 41.7%, 47.0%, 45.7%, 40.9%, and 41.2%, and the abundance ratios of Bacteroideae were 31.9%, 28.5%, 31.6%, 31.9%, 32.4%, and 31.5%, respectively. The abundance ratios of selenomonas were 12.8%, 10.8%, 11.9%, 12.4%, 11.6%, 11.0%, and enterobacteriaceae were 5.7%, 5.2%, 5.3%, 4.6%, 4.1%, 5.1%, respectively. The abundance ratios of bifidobacteria were 1.9%, 8.9%, 1.2%, 2.3%, 2.6%, 5.3%, respectively (see Figure 5).

**FIGURE 6:**
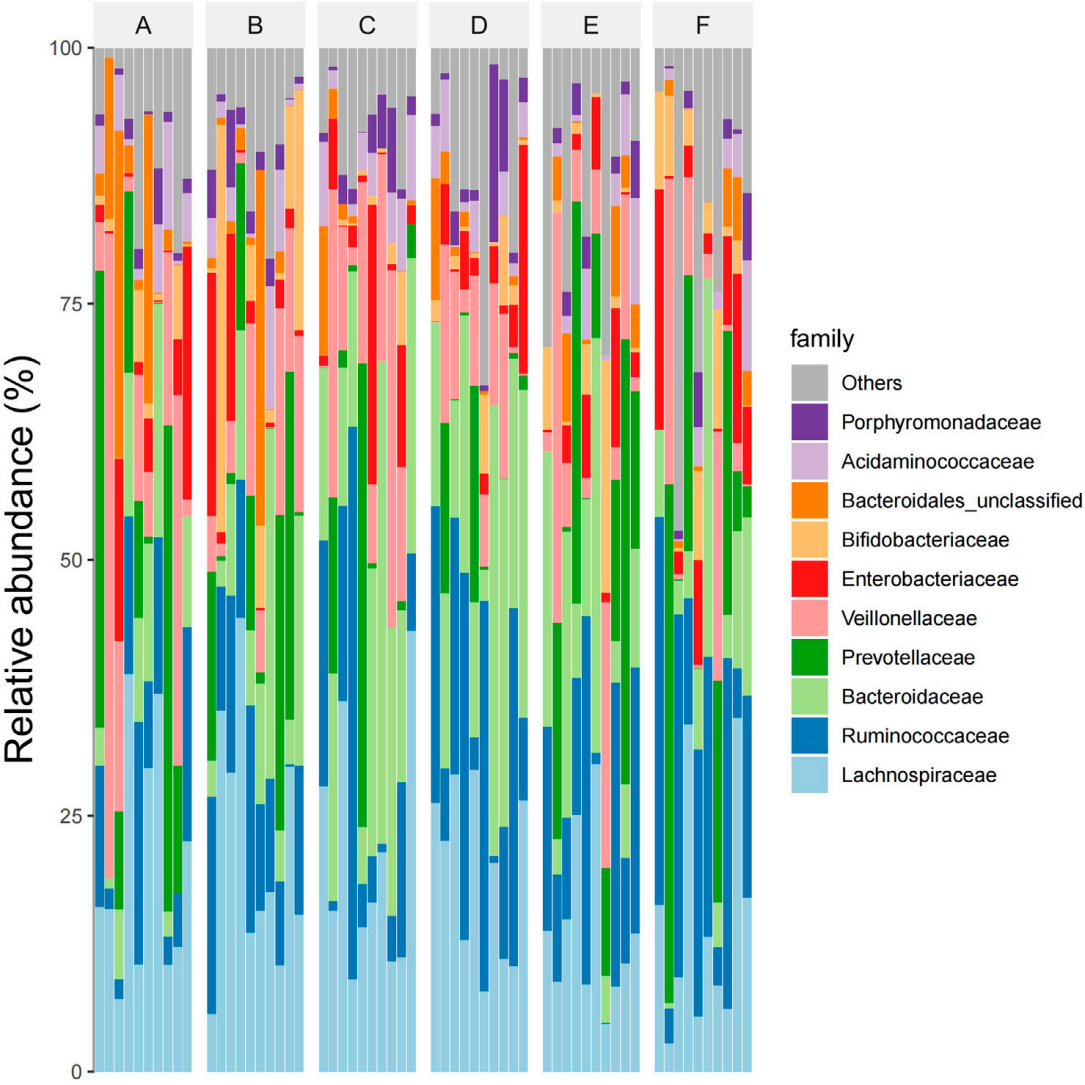
Abundations of species composition in six groups of samples at family level (Note: Ibid).

At the subject level, The dominant intestinal bacteria in the six groups were Lachnospiraceae, Ruminococcaceae, Bacteroidaceae, Prevotellaceae and Veillonellaceae), Enterobacteriaceae, Bifidobacteriaceae, Bacteroidiales-unclassified, Acidaminococcaceae, and Cyanobacteria Porphyromonadaceae. The abundance ratios of Trichospirillaceae in groups A, B, C, D, E, and F were 24.6%, 24.8%, 17.8%, 17.1%, 17.4%, and 19.3%, and that of ruminaceae were 8.0%, 9.2%, 11.1%, 14.3%, 12.4%, and 14.1%, respectively. The abundance ratios of Bacteroideaceae were 9.2%, 12.2%, 17.7%, 18.0%, 12.7%, 10.3%, and Prevotellaceae were 10.6%, 11.5%, 8.1%, 6.9%, 13.5%, 11.3%, respectively. The abundance ratios of Veronaceae were 12.7%, 10.7%, 11.3%, 8.9%, 9.5%, 7.9%, and Enterobacteriaceae were 5.7%, 5.2%, 5.3%, 4.6%, 4.3%, 4.8%, respectively. The abundance ratios of Bifidobacteriaceae were 1.9%, 8.9%, 1.2%, 2.3%, 1.6%, 1.9%, and the abundance ratios of unclassified Bacteroideae were 5.2%, 4.3%, 1.1%, 0.9%, 1.3%, 0.7%, respectively. The abundance ratios of Aminococcaceae were 2.1%, 2.3%, 2.4%, 1.8%, 1.7%, 1.4%, and that of violomonas family were 0.9%, 1.1%, 2.1%, 2.8%, 1.2%, 0.7%, respectively (see Figure 6).

**FIGURE 7:**
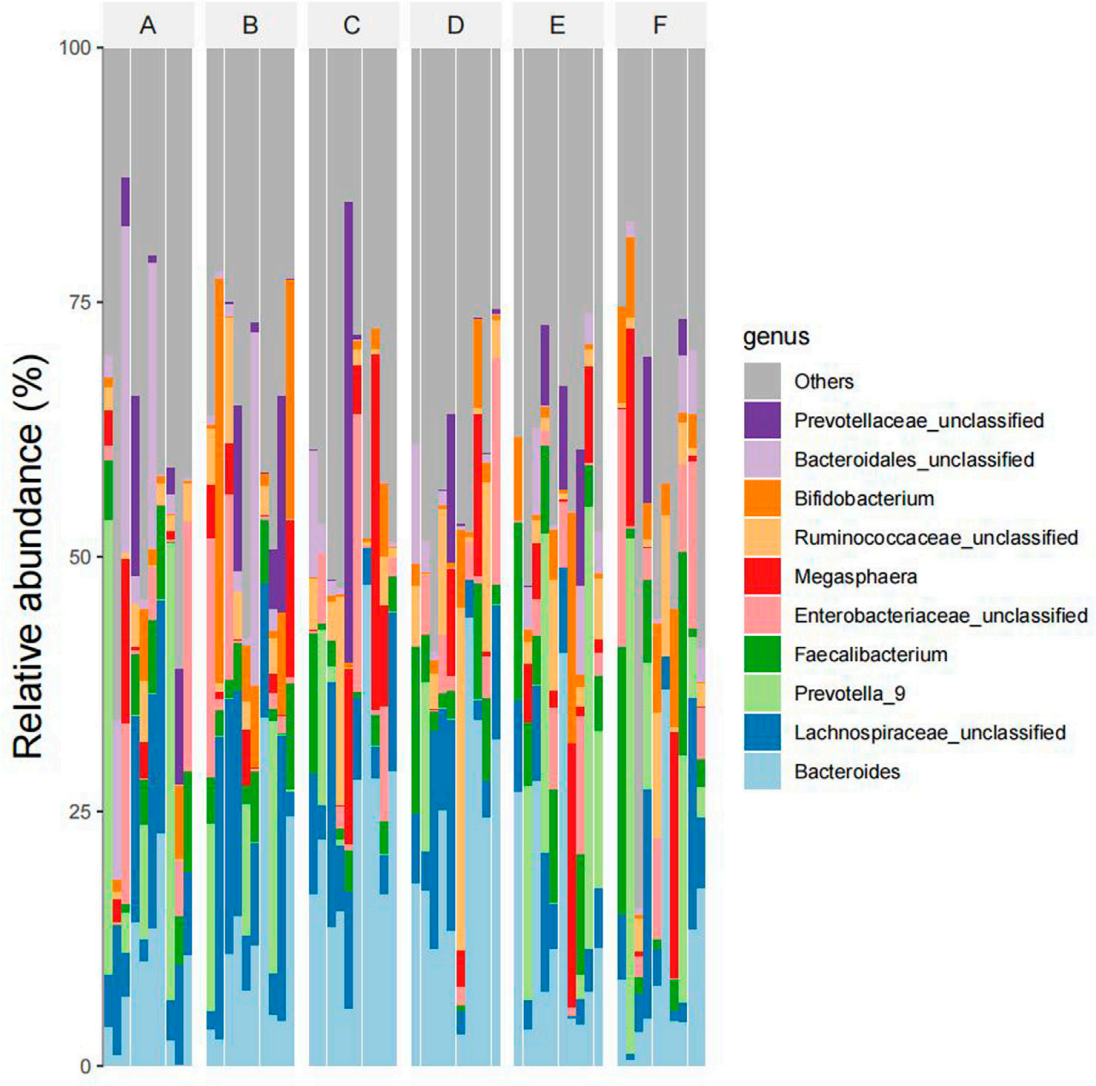
Abundances of species composition in six groups of samples at the generic level (Note: Ibid).

At the generic level, The dominant bacteria in the intestinal tract of the four groups were *Bacteroides*, Lachnospira-unclassified, Prevotella-9, Faecalibacterium and *Enterobacter* obacteriaceae-unclassified, Megasphaera, Ruminococcaceae-unclassified, Bifidobacterium, Bact eroidaceae-unclassified, Prevotellaceae-unclassified genus. The abundance ratios of *Bacteroides* in groups A, B, C, D, E, and F were 12.8%, 13.1%, 21.6%, 21.8%, 15.6%, and 12.3%, respectively. The abundance ratios of unclassified genera of Trichomillaceae were 12.7%, 11.7%, 6.5%, 5.9%, 3.8%, and 6.4%, respectively. The abundance ratios of Prevotella were 9.7%, 8.5%, 2.1%, 1.7%, 15.3%, and 16.5%, and that of Faecalis were 5.9%, 5.4%, 4.2%, 4.6%, 5.2%, and 4.5%, respectively. The abundance ratios of *enterobacter* unclassified genera were 5.2%, 5.4%, 3.5%, 2.7%, 4.2%, and 5.8%, and the abundance ratios of Macrococcus genera were 2.1%, 2.9%, 4.3%, 3.9%, 4.7%, and 3.1%, respectively. The abundance ratios of unclassified rumen bacteria were 2.2%, 2.6%, 3.7%, 5.4%, 2.4%, 2.8%, and bifidobacteria were 2.2%, 5.8%, 1.6%, 1.9%, 3.6%, 4%, respectively. The abundance ratios of unclassified genera of Bacteroideaceae were 3.1%, 2.1%, 0.9%, 0.6%, 1.4%, 0.8%, and those of unclassified genera of Prevotella family were 4.1%, 3.0%, 4.5%, 1.1%, 2.9%, 1.8%, respectively (see Figure 7).

#### 3.6.2 Generic level based clustering heat map

**FIGURE 8:**
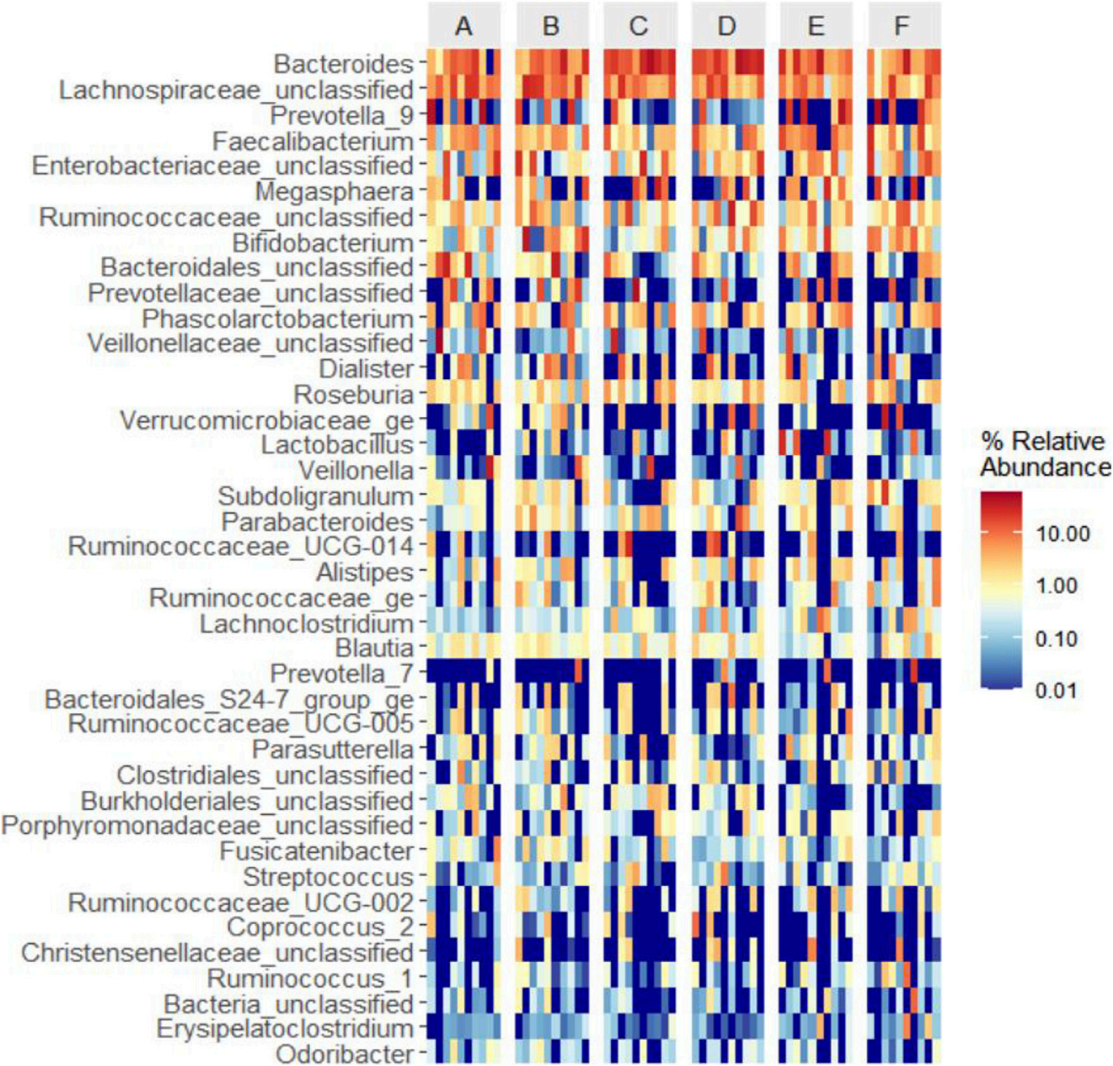
Clustering heat map analysis of six groups of samples at the generic level (Note: Ibid).

Based on the species abundance information of the samples at the genus level, the top 40 genera of abundance were selected, and the samples and species were clustered based on the abundance information of each sample and plotted in a heatmap (Figure 8), which makes it easy to observe the similarity between the samples as well as the similarity of the community composition at the genus level.

#### 3.6.3 Beta diversity analysis and inter-group difference analysis

**FIGURE 9:**
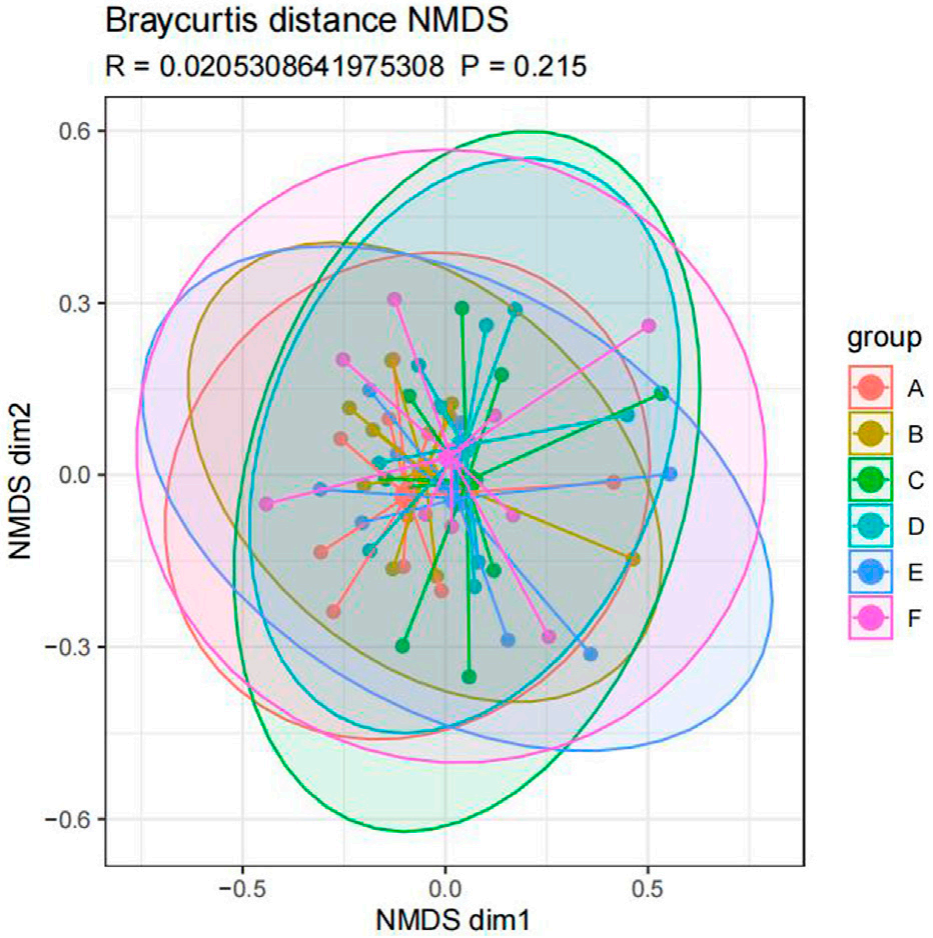
Beta diversity of intestinal flora in six groups of patients (Note: Ibid).

**FIGURE 10:**
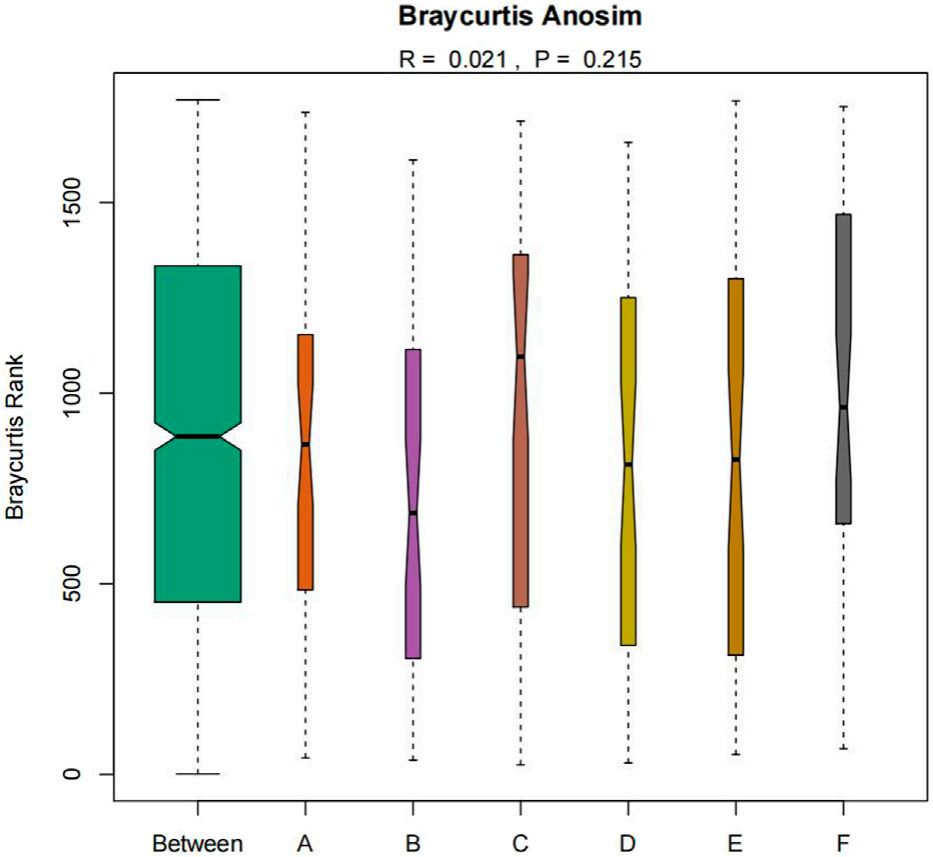
Anosim test of intestinal flora in six groups of patients (Note: Ibid).

The similarity of microbial communities can be observed by Nonmetric Multidimensional Scaling (NMDS). Every point in the figure represents a sample. Points with the same color can be found in the same group. The closer the distance between two points, the smaller the difference in community composition between the two points (Figure 9). Anosim test was used to test whether the difference between groups was significantly greater than the difference within groups (Figure 10). R value = 0.021 > 0 indicated that the difference between groups was greater than the difference within groups, and *p*-value = 0.215 > 0.05 indicated that the difference was not significant. There was no significant qualitative difference in intestinal microbial community structure among the six groups, indicating that there was no significant qualitative difference in intestinal flora between the Jin Gui Ren Qi Pill group, *Bifidobacterium bifidum* tetragonum tablets group and control group before and after intervention and between groups.

#### 3.6.4 LEfSe analysis

**FIGURE 11:**
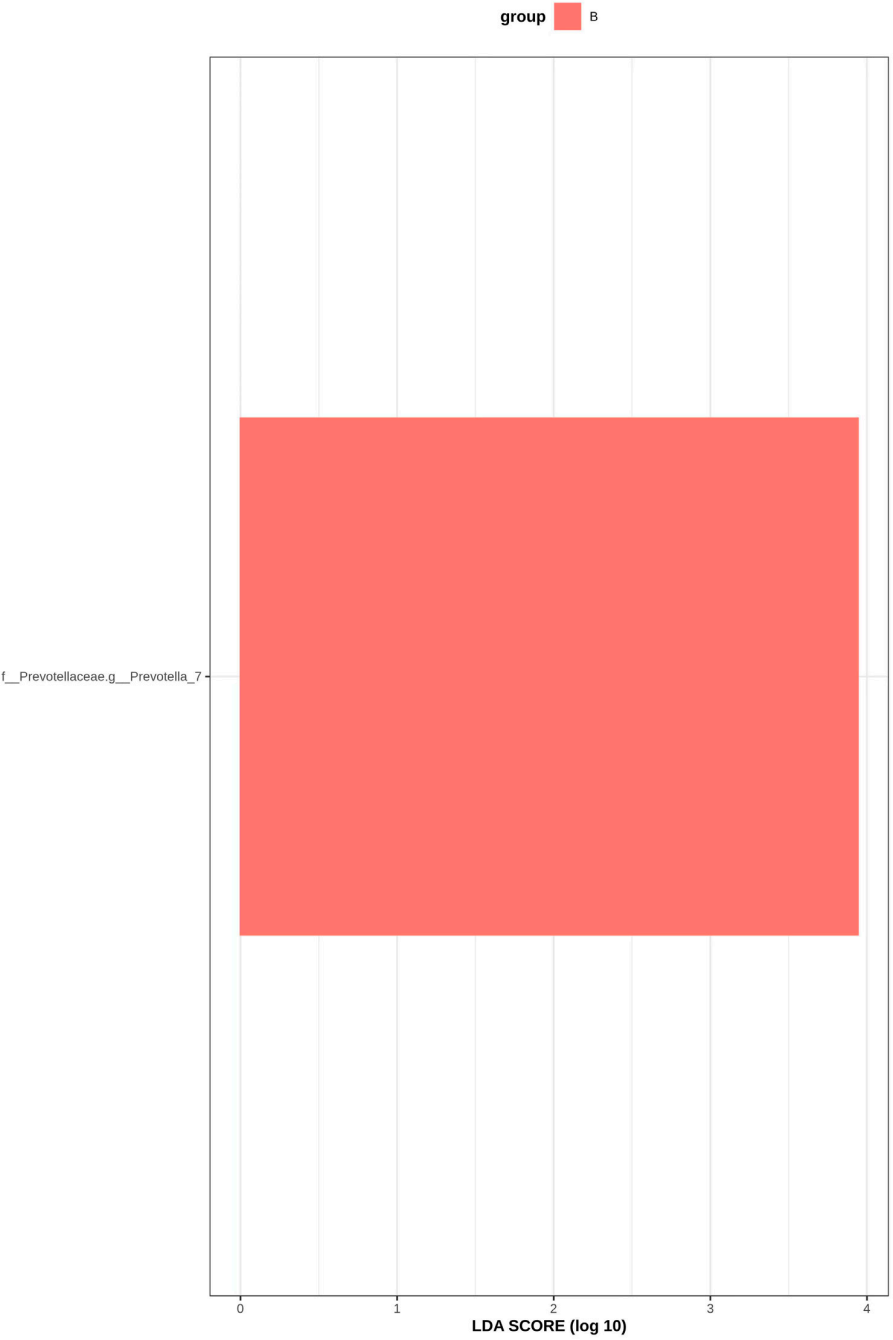
Histogram of LDA value distribution.

LEfSe analysis is an analytical method based on linear discriminant analysis (LDA) that can be used to identify important species with significant differences between groups. Through LDA analysis, significant bacteria genera were identified in the six groups, and the LDA score with significant differences was set at 2, *p* < 0.05 (Krukal Wallis Anova test and Wilcoxon test). Figure 11 shows the histogram of LDA value distribution: The horizontal coordinate is the LDA value, that is, the influence degree of significantly different species between different groups; the vertical axis is the significantly different species, and different colors represent different groups. According to the results, f_Prevotellaceae.g_Prevotella_7 bacteria increased in group B, indicating that the abundance of Prevotella_7 in GM patients with DKD increased after the intervention of Jin Gui Ren Qi Pill.

#### 3.6.5 Random forest analysis

**FIGURE 12:**
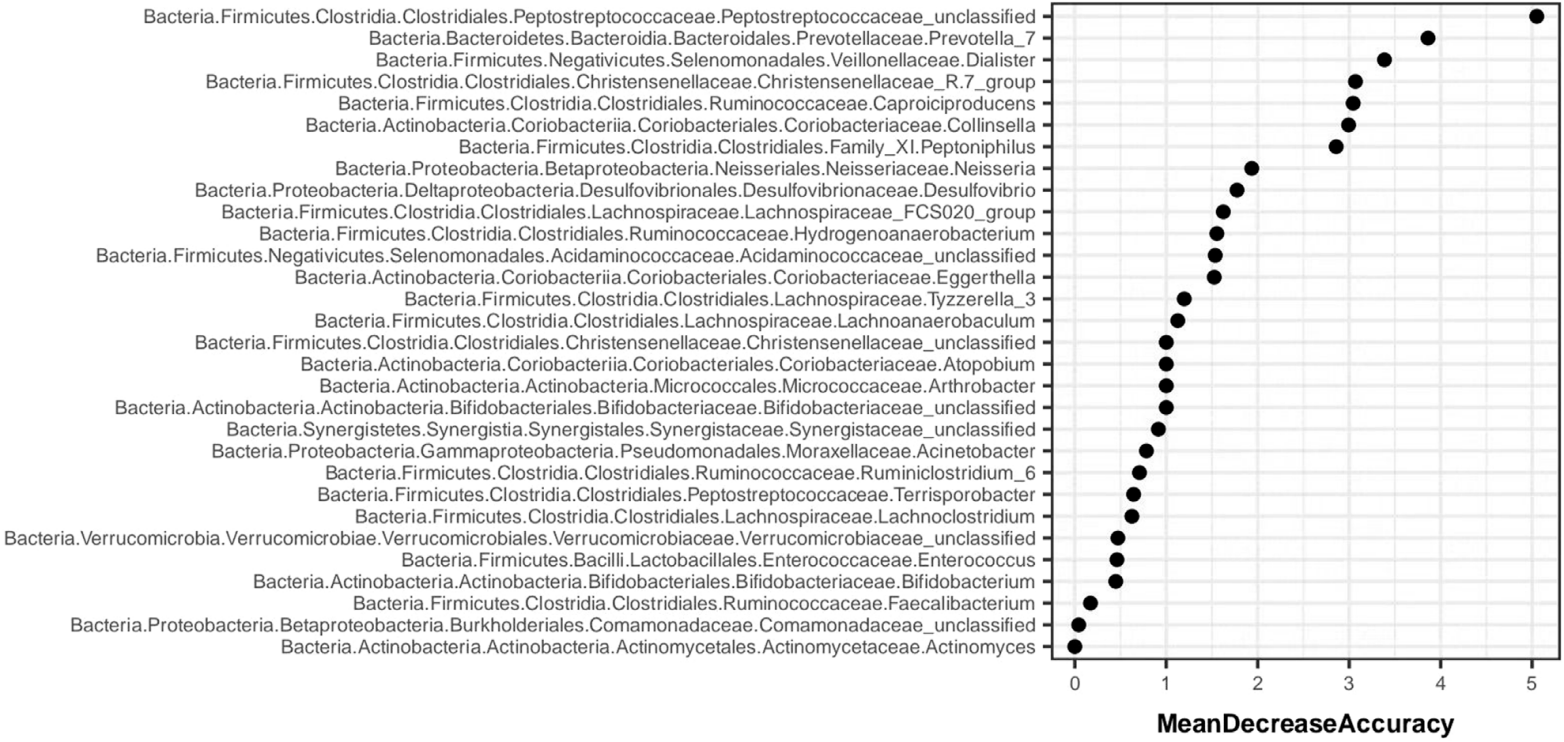
Species importance table.

Random forest analysis is implemented through the “randomForest” package of R language. Figure 12 shows the species importance table of random forest analysis based on species abundance. MeanDecreaseAccuracy is a measure of changing the value of a variable into a random number, and the random number represents the degree of reduction in the prediction accuracy of random forest. The greater the MeanDecreaseAccuracy, the greater the importance of the variable. According to the species importance table, Prevotella_7 was ranked the second.

#### 3.6.6 KEGG function prediction

**FIGURE 13:**
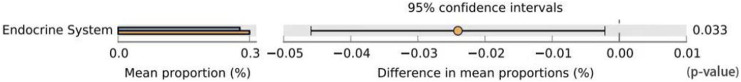
Prediction of GM function difference between group A and group B. Note: Group A: pre-intervention with Jin Gui Ren Qi Pill, Group B: post-intervention with Jin Gui Ren Qi Pill.

**FIGURE 14:**
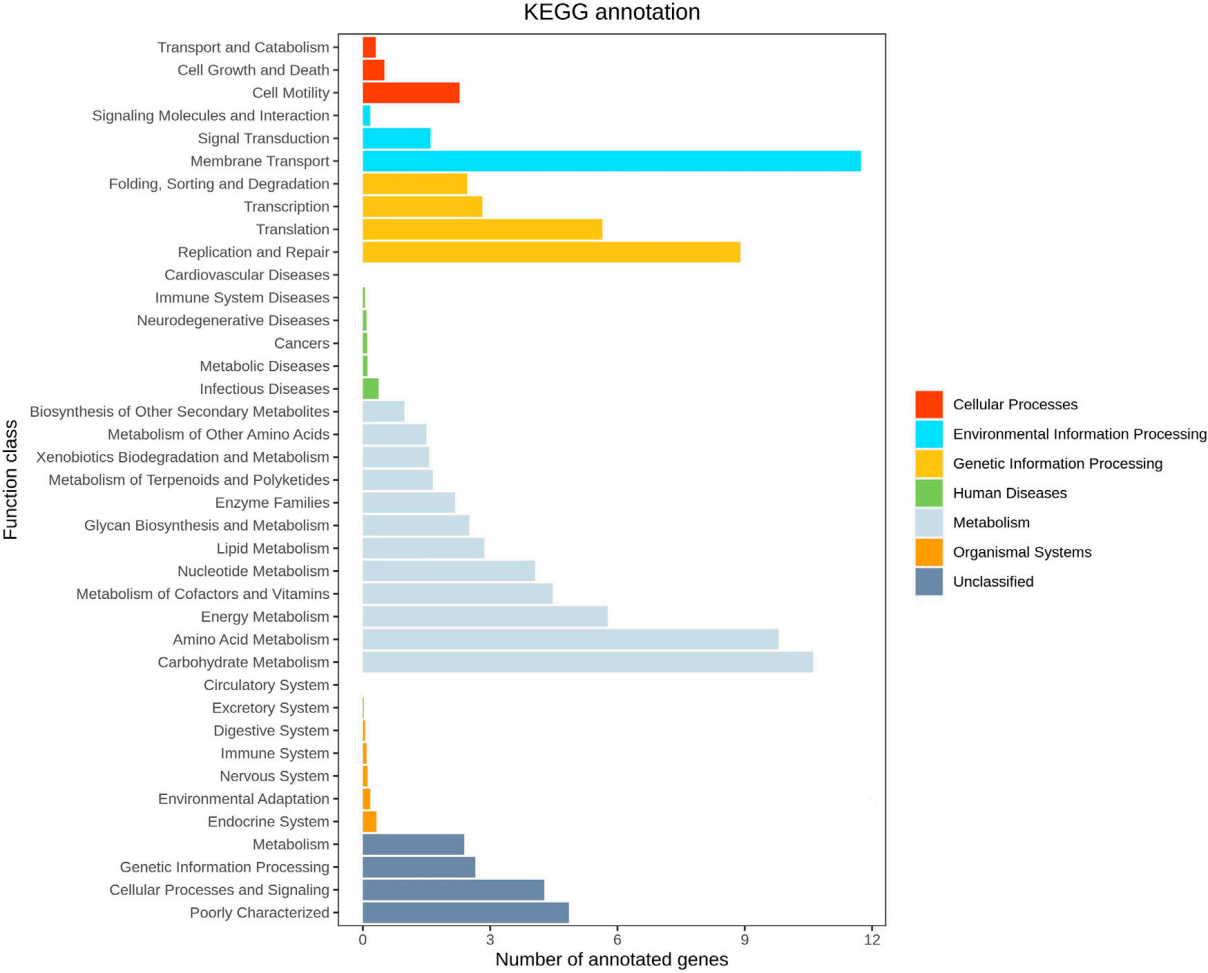
KEEG function prediction chart.

Functional prediction was conducted utilizing 16S sequencing data to discern functional variances among different samples and groups. Leveraging the 16S rDNA information of tested bacteria and the OTU data of closely related species in the Greengenes database, PICRUSt software primarily inferred the gene function spectrum of their common ancestors. Simultaneously, it predicted the gene function spectrum of other untested species in the Greengenes database. This process constructed the gene function prediction spectrum of archaea and the entire lineage of bacterial domains. Subsequently, the sequenced bacterial community composition was “mapped” to the database to predict the metabolic function of the bacterial community. Comparison between groups AB revealed a notable increase in the level of carbohydrate metabolism in group B (*p* = 0.038 < 0.05), indicating enhanced carbohydrate metabolism function in patients treated with Jin Gui Ren Qi Pill (Figure 13). A histogram was generated based on the resultant prediction outcomes of KEGG second-level metabolic pathways (Figure 14). The horizontal axis depicted the relative abundance of annotated genes, while the vertical axis denoted the names of the second-level functions. Various colors in the graph corresponded to the first-level functions of the second-level, with the abundance of genes linked to carbohydrate metabolism ranking second.

### 3.7 Correlation analysis between intestinal flora of patients and clinical characteristics and indicators

**FIGURE 15:**
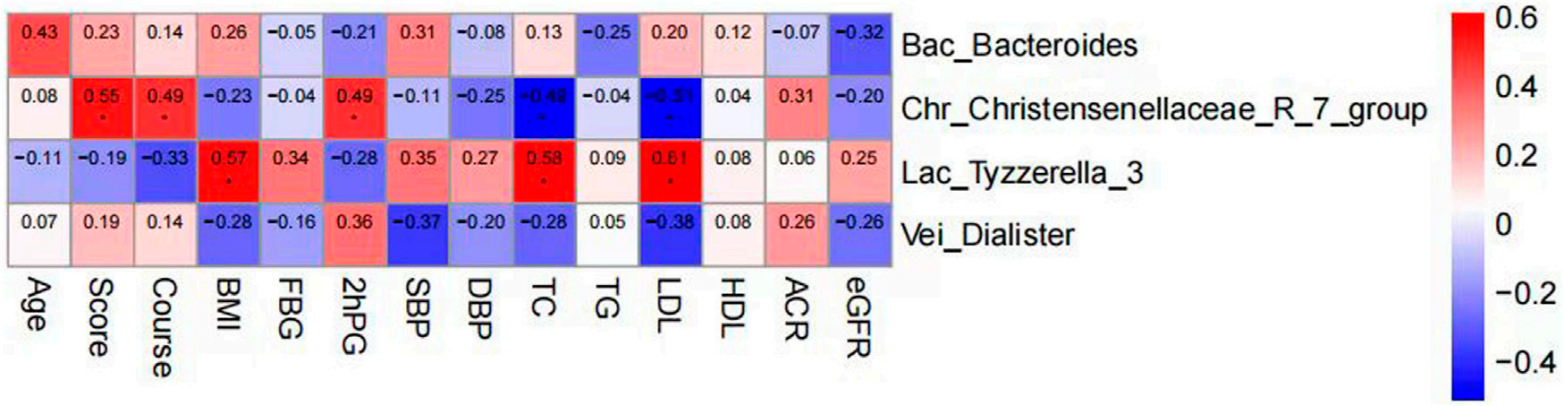
Clustering heat map analysis between intestinal flora and clinical features and indicators in DKD patients.

Spearman correlation analysis was employed to delve deeper into the relationship between intestinal flora and clinical features and indicators in DKD patients. Our findings revealed that BMI, TC, and LDL exhibited positive correlations with the relative abundance of Lac_Tyzzerella_3 flora. Moreover, TCM syndrome score, age, duration of disease, and 2hPBG (2hPG) after meals were positively correlated with the relative abundance of Chr_Christensenellaceae_R_7_group. Conversely, TC and LDL showed negative correlations with the relative abundance of Chr_Christensenellaceae_R_7_group bacteria (Figure 15).

## 4 Discussion

Contemporary theoretical and clinical investigations have underscored the significant role of dysbiosis stemming from alterations in the intestinal microecological structure and diversity in the progression of diabetes mellitus and diabetic kidney disease (Fernandez-Prado et al., 2017). Even in the early stages of DKD, the composition of the gut flora undergoes changes (Andrade-Oliveira et al., 2015). In the current study, we aimed to assess the impact of adjunctive therapy with Jin Gui Ren Qi Pill and *Bifidobacterium bifidum* tetragonum tablets on glycemic control, blood pressure regulation, lipid metabolism, and enhancement of renal function in individuals afflicted with diabetic nephropathy. After a 4-week intervention, we observed significant differences among the treatment groups. Specifically, the Jin Gui Ren Qi Pill group exhibited notably superior improvements in ACR compared to the other two groups, with a significant correlation evident (*p* < 0.05). Conversely, the *Bifidobacterium bifidum* tetragonum tablets group outperformed the other two groups in terms of TC improvement post-intervention, with a significant correlation noted (*p* < 0.05). Notably, there were no significant differences in blood pressure changes before and after the intervention across the three groups. Overall, the Jin Gui Ren Qi Pill group demonstrated greater improvements in TCM evidence scores and ACR compared to the other groups, albeit not matching the *Bifidobacterium bifidum* tetragonum tablets group’s efficacy in improving glucose and lipid metabolism. Conversely, the *Bifidobacterium bifidum* tetragonum tablets group exhibited significant enhancements in blood lipid metabolism compared to the other groups, albeit with slightly less pronounced improvements in glucose metabolism and creatinine levels. Compliance in this study was good overall. There was one case of diarrhea in the *Bifidobacterium bifidum* tetragonum tablets group, which resolved with treatment. This indicates a potential risk for adverse events associated with this therapy.

In TCM, DKD falls under the category of “thirst-quenching kidney disease,” characterized by kidney yang deficiency, yin deficiency, dryness, heat, weakened qi, lumbar region malnourishment, and diminished ability to intake and retain substances, leading to the depletion of both yin and yang (Haiyang and Zhang, 2012; Fang et al., 2016). “Jin Gui Kidney Qi Pill,” as documented in “The Essentials of the Golden Chamber,” is prescribed to invigorate the kidney and reinforce yang, facilitate water metabolism, and balance yin and yang energies. It is indicated for addressing symptoms such as phlegm, stasis, and excessive thirst due to deficiencies in qi, blood, yin, and yang. Several research studies have demonstrated the efficacy of Jin Gui Ren Qi Pill in ameliorating impaired serum glucose metabolism levels and renal function in patients with diabetic nephropathy (Liu, 2014; Jin, 2019; Yuan and Nie, 2020). In this study, Jin Gui Ren Qi Pill demonstrated a more significant improvement in the TCM evidence score and ACR compared to the control group. However, there were no significant differences observed in the metabolism of blood glucose, blood lipids, and creatinine. Several factors may contribute to these findings: 1. The conventional treatment with western medicine may mask the effects of Jin Gui Ren Qi Pill on glucose and lipid metabolism. 2. The fixed components of the Chinese medicine reagents used in this experiment do not allow for flexible adjustment according to individual patient needs. 3. The adjustment of prescription drugs did not fully leverage the advantages of evidence-based Chinese medicine treatment. 4. Single measurements of FBG and 2hPBG may not adequately reflect blood glucose control. Dynamic monitoring of serum glucose levels could provide a more comprehensive assessment. Additionally, factors such as diet and exercise may have influenced blood glucose levels during the experiment. 5. The small sample size of this study increases the likelihood of bias in the results.

Aberrant lipid metabolism is intricately linked with the mortality rates associated with atherosclerosis, cardiovascular diseases, and cerebrovascular diseases (Afsharian et al., 2016). Shimizu’s research demonstrated that dysregulated lipid metabolism can emerge as a potent independent predictor of ESRD progression (Shimizu et al., 2015), while findings underscored its significance as a key contributor to the advancement of diabetic nephropathy (Huang, 2019). This study corroborated the efficacy of *Bifidobacterium bifidum* tetragonum tablets in ameliorating total cholesterol levels among DKD patients, aligning with the consensus across most investigations. This effect could be attributed to the augmentation of SCFAs, which in turn inhibit the hepatic synthesis of cholesterol and fatty acids, facilitate cholesterol clearance, and impede enzymatic cholesterol synthesis (Liu et al., 2019). Ehrlich et al. (2020) discovered that indole-3-lactic acid (ILA), the primary tryptophan catabolic product generated by Bifidobacterium *in vitro*, possesses the capability to diminish the production of TNF-α and the pro-inflammatory factor IL-8 induced by lipopolysaccharide (LPS), thereby mitigating the inflammatory cascade. Similarly, Zhao et al. (2021) elucidated that bifidobacterium can notably downregulate the expression of LPS-induced TNF-α, IL-1β, and IL-6 mRNA, attenuate the inflammatory response, and shield against LPS-induced intestinal barrier damage. Building upon these insights, this study delved further into the impact of *Bifidobacterium bifidum* tetragonum tablets on inflammatory factors. The investigation revealed that compared to the control group, the intervention of *Bifidobacterium bifidum* tetragonum tablets significantly reduced the IL-2 content in DKD patients, with statistical significance (*p* < 0.05), corroborating findings from the majority of prior research. However, there was no significant difference observed in the suppression of IL-6 and TNF-α in the *Bifidobacterium bifidum* tetragonum tablets group post-intervention compared to the control group, contrary to earlier studies. The disparities between this study and previous ones were primarily attributed to the following factors: Firstly, the duration of medication in this study was relatively short, and the enhancement of intestinal flora and successful colonization of beneficial bacteria typically require a longer timeframe. Consequently, the improvements in impaired serum glucose metabolism and kidney function may not have been readily apparent. Secondly, the fluctuation of blood glucose and inflammatory factors in patients can be significantly influenced by dietary habits, physical activity, and stress levels, thereby introducing potential confounding variables into the experiment. Lastly, the small sample size of this study increases the likelihood of bias, underscoring the necessity to expand the sample size for more robust experimental findings.

In this study, 16S rDNA high-throughput sequencing technology was employed to analyze the genes of fecal intestinal flora in patients both before and after intervention, facilitating an examination of the alterations in intestinal flora. Research findings suggest that, at the phylum level, the dominant bacteria in healthy individuals in Shanxi are Firmicutes (52.52%), Bacteroidetes (42.87%), and Proteobacteria (2.76%) (Qi et al., 2019). The abundance of Bacteroidetes (35.9%, 33.5%, 35.6%, 35.9%, 37.1%, 29.8%), and Proteobacteria (7.6%, 7.1%, 8.0%, 5.7%, 7.5%, 6.7%) in the intestinal flora of DKD patients collected in this study was found to be lower compared to that of healthy individuals, yet higher than the healthy population in general. These shifts in dominant flora were consistent with previously reported alterations in intestinal flora among DKD patients (Rizzatti et al., 2017; Feng et al., 2020). Bacteroidetes, as the predominant anaerobic gram-negative bacterium in the human intestine, typically offers beneficial effects, aiding in the breakdown of complex polysaccharides in the intestine for energy provision, regulation of the human immune system, and prevention of pathogen colonization (Zafar and Saier, 2021). Proteobacteria, a gram-negative bacterium, demonstrates increased abundance in the intestinal tract of DKD patients. This elevation in abundance contributes to bacterial translocation, thereby elevating the circulating levels of lipopolysaccharide (LPS). Consequently, it activates the LPS-TLR2/TLR4-NF-κB signaling pathway, triggering the release of inflammatory factors (TNF-α, IL-1, IL-6, etc.), thus perpetuating long-term inflammation. This inflammatory state can lead to insulin resistance and kidney damage. This study reaffirms the presence of disturbances in intestinal flora among DKD patients. Notably, there was no significant difference observed in GM α and β diversity between the Jin Gui Ren Qi Pill group, *Bifidobacterium bifidum* tetragonum tablets group, and the control group before and after intervention. This suggests that there were no substantial alterations in GM abundance, diversity, and heterogeneity between the groups pre- and post-intervention. Possibly due to: 1. Insufficient intervention time: Probiotic colonization necessitates a prolonged, stable environment. The intervention period in this experiment may have been too short to establish a new homeostatic balance in the intestines. 2. Small sample size: With a limited number of participants, there is a heightened probability of bias in the results. Nevertheless, we maintain the hypothesis that even a short-term (4 weeks) intervention can exert some influence on the homeostasis of intestinal flora in DKD patients. Through LEfSe analysis, we identified distinct bacterial genera following the intervention with Jin Gui Ren Qi Pill. This finding suggests a gradual disruption of the old intestinal flora homeostasis and a transition towards a new equilibrium. After treatment with Jin Gui Ren Qi Pill, there was an increase in the abundance of Prevotella_7 in the intestines of DKD patients, with Prevotella_7 ranking second according to the species importance table generated by statistical analysis. Prevotella is an anaerobic gram-negative bacterium and a commensal organism in the human gut. The beneficial effects of Prevotella on human health are still debated (Tett et al., 2021). On one hand, Kovatcheva-Datchary et al. (2015) demonstrated that dietary fiber supplementation increased the abundance of intestinal Prevotella copri, which had beneficial effects on glucose metabolism in some healthy individuals. On the other hand, Prevotella copri promotes the synthesis of branched-chain amino acids (BCAAs), and elevated serum levels of BCAAs are characteristic of insulin-resistant serum metabolites in nondiabetic patients, suggesting that the enrichment of Prevotella copri may be related to insulin resistance (Pedersen et al., 2016). Additionally, Prevotella spp. are enriched in the intestines of individuals with prehypertension, essential hypertension, and those at high risk for cardiovascular disease, suggesting that Prevotella spp. may increase the lifelong risk of cardiovascular disease (Kelly et al., 2016; Li et al., 2017). Based on KEGG function prediction, carbohydrate metabolism in the intestinal flora was enhanced in patients treated with Jin Gui Ren Qi Pill, effectively corroborating the findings of Kovatcheva-Datchary et al. (2015). This suggests a more favorable outlook for the adjuvant treatment of DKD with Jin Gui Ren Qi Pill.

Spearman correlation analysis was utilized to further investigate the relationship between changes in intestinal flora and clinical indicators in DKD patients. The analysis revealed that BMI, TC, and LDL were positively correlated with the relative abundance of Tyzzerella_3 flora, suggesting a potential link between the enrichment of Tyzzerella_3 and obesity and dyslipidemia, consistent with prior research findings. Conversely, TC and LDL exhibited a negative correlation with the relative abundance of Christensenellaceae_R_7 flora. Research by Waters et al. (Waters and Ley, 2019) indicated that Christensenellaceae, a member of Firmiculus, is one of the five groups associated with a healthy gut microbiota profile. It is inversely correlated with various markers of adiposity, dyslipidemia, iimpaired serum glucose metabolism, and blood pressure, making it a promising probiotic candidate for improving human health, aligning with the outcomes of our study. The positive correlation observed between age, duration of disease, postprandial blood glucose, and Christensenellaceae_R_7 warrants further investigation and discussion.

Through studying the effects of Jin Gui Ren Qi Pill and *Bifidobacterium bifidum* tetragonum tablets on the intestinal flora and metabolism of DKD patients, we found that the human intestinal flora remains relatively stable over a short period (4 weeks) with an unchanged diet and exercise regimen. But Jin Gui Ren Qi Pill increased the abundance of Prevotella_7 in the gut microbiota of DKD patients and enhanced the carbohydrate metabolism capabilities of intestinal bacteria. This study fills a gap in understanding how Jin Gui Ren Qi Pills affect the intestinal flora of DKD patients, providing a new direction for exploring their mechanism of action and addressing the limitations of previous research. Meanwhile, Jin Gui Ren Qi Pill has obvious improvement on TCM evidence and kidney function. The improvement of lipid metabolism by *Bifidobacterium bifidum* tetragonum tablets is relatively significant. Additionally, these tablets effectively reduce IL-2 levels in DKD patients, thereby improving oxidative stress and inflammation. This supports our hypothesis that this established microbial preparation can effectively reduce inflammation in DKD patients and may potentially be used to delay the progression of DKD over the long term. In conclusion, this study provides strong evidence for the combined use of traditional Chinese medicine, Western medicine, and probiotics in treating DKD with yin and yang deficiency, offering new insights for DKD treatment.

In our study, several research limitations need to be acknowledged. Firstly, due to constraints in time and manpower, our sample size remained relatively small, potentially introducing bias that could influence the outcomes, and thus limiting the generalizability of our findings. Moreover, we were unable to explore the comprehensive effects of Jin Gui Ren Qi Wan and *Bifidobacterium bifidum* tetragonum tablets on DKD, as we did not assess alterations in patients’ gut microbiota metabolites and SCFAs before and after the intervention. This hampers our ability to fully elucidate the mechanisms underlying the observed effects of the interventions. Additionally, the short follow-up period limited the observation of significant long-term changes in the gut microbiome and clinical outcomes.

In conclusion, the integration of Jin Gui Ren Qi Pill with conventional Western medical therapy exhibits superior efficacy in ameliorating clinical symptoms and reducing ACR in DKD patients compared to Western medical treatment alone. This combined approach also enhances the abundance of intestinal flora Prevotella_7 in patients, potentially augmenting carbohydrate metabolism by the intestinal flora. Furthermore, the supplementation of *Bifidobacterium bifidum* tetragonum tablets alongside Western medical treatment demonstrates enhanced reduction in TC levels in DKD patients compared to Western medicine treatment alone. Moreover, it effectively reduces IL-2 content and inflammatory levels in patients, showcasing promising anti-inflammatory effects.

## Data Availability

The original contributions presented in the study are included in the article/Supplementary Material, further inquiries can be directed to the corresponding author.

## Abbreviations

FBG, fasting blood glucose; 2hPBG, 2 hours postmeal blood glucose; TG, triglyceride; TC, serum total cholesterol; LDL-C/LDL, serum low density lipoprotein cholesterol; HDL-C, serum high density lipoprotein cholesterol; ACR, random urinary microalbumin/creatinine ratio; SCr, serum creatinine; ELISA, Enzyme-linked-Immuno-Sorbent Assay; DKD, diabetic kidney disease; TCM, traditional chinese medicine.

## References

[B1] Afsharian S. Akbarpour S. Abdi H. Sheikholeslami F. Moeini A. S. Khalili D. (2016). Risk factors for cardiovascular disease and mortality events in adults with type 2 diabetes - a 10-year follow-up: tehran Lipid and Glucose Study. Diabetes Metab. Res. Rev. 32 (6), 596–606. 10.1002/dmrr.2776 26787367

[B2] Alicic R. Z. Rooney M. T. Tuttle K. R. (2017). Diabetic kidney disease: challenges, progress, and possibilities. Clin. J. Am. Soc. Nephrol. 12 (12), 2032–2045. 10.2215/CJN.11491116 28522654 PMC5718284

[B3] Andrade-Oliveira V. Amano M. T. Correa-Costa M. Castoldi A. Felizardo R. J. F. de Almeida D. C. (2015). Gut bacteria products prevent AKI induced by ischemia-reperfusion. J. Am. Soc. Nephrol. 26 (8), 1877–1888. 10.1681/ASN.2014030288 25589612 PMC4520159

[B4] Aoki R. Kamikado K. Suda W. Takii H. Mikami Y. Suganuma N. (2017). A proliferative probiotic Bifidobacterium strain in the gut ameliorates progression of metabolic disorders via microbiota modulation and acetate elevation. Sci. Rep. 7, 43522. Published 2017 Mar 2. 10.1038/srep43522 28252037 PMC5333160

[B5] Arora M. K. Singh U. K. (2013). Molecular mechanisms in the pathogenesis of diabetic nephropathy: an update. Vasc. Pharmacol. 58 (4), 259–271. 10.1016/j.vph.2013.01.001 23313806

[B6] Cani P. D. Bibiloni R. Knauf C. Waget A. Neyrinck A. M. Delzenne N. M. (2008). Changes in gut microbiota control metabolic endotoxemia-induced inflammation in high-fat diet-induced obesity and diabetes in mice. Diabetes 57 (6), 1470–1481. 10.2337/db07-1403 18305141

[B7] Chen X. Zhang L. J. Wu Z. (2019). Meta-analysis of therapeutic effect of Jinkui Shenqi pills on diabetic nephropathy. Jilin Tradit. Chin. Med. (2), 5.

[B8] Dai X. X. Hong-dieSushulan CAIH. (2017). Regulatory effects of Rehmannia leaf on intestinal flora in rats with diabetic nephropathy. Acta Pharmacol. Sin. 52 (11), 1683–1691.

[B9] Diabetes Society (2021). Chinese Medical Association. Chinese Guidelines for the prevention and treatment of type 2 diabetes (2020 edition). Int. J. Endocrinol. Metabolism 41 (05), 482–548.

[B10] Ehrlich A. M. Pacheco A. R. Henrick B. M. Taft D. Xu G. Huda M. N. (2020). Indole-3-lactic acid associated with Bifidobacterium-dominated microbiota significantly decreases inflammation in intestinal epithelial cells. BMC Microbiol. 20 (1), 357. Published 2020 Nov 23. 10.1186/s12866-020-02023-y 33225894 PMC7681996

[B11] Expert Group of Chinese Society of Nephrology (2021). Chinese guidelines for clinical diagnosis and treatment of diabetic kidney disease. Chin. J. Nephrol. 37 (3), 50. (in Chinese).

[B12] Fang Y. Yang Y. Yan S. (2016). Theoretical framework of etiology and pathogenesis of diabetic nephropathy. J. Liaoning Univ. Traditional Chin. Med. 18 (11), 53–55. (in Chinese).

[B13] Feng C. N. Zeng L. Z. Wang S. J. (2020). Patients with type 2 diabetes mellitus and diabetic nephropathy. Analysis of inflammation and intestinal microbial diversity. Chin. J. Microecology 32 (11), 1273–1278. (in Chinese).

[B14] Fernandez-Prado R. Esteras R. Perez-Gomez M. V. Gracia-Iguacel C. Gonzalez-Parra E. Sanz A. B. (2017). Nutrients turned into toxins: microbiota modulation of nutrient properties in chronic kidney disease. Nutrients 9 (5), 489. Published 2017 May 12. 10.3390/nu9050489 28498348 PMC5452219

[B15] Guoliang K. Jin S. Yang Z. (2021). Effect of bifidobacterium capsules in type 2 diabetes with gastrointestinal dysfunction. Henan Med. Res. 30 (17), 3192–3194.

[B16] Haiyang L. Zhang C. (2012). Research progress on etiology and pathogenesis of Xiaoke nephropathy. Chin. J. Integr. Traditional West. Nephrop. 13 (01), 84–85. (in Chinese).

[B17] Huang L. H. Xiang S. (2019). Research progress of Jinkui Shenqi Pill in treating diabetic nephropathy. Popular Sci. Technol. 21 (11), 4.

[B18] Huang Y. W. (2019). Analysis of the relationship between diabetic nephropathy and abnormal lipid metabolism. New World Diabetes Mellit. 23 (06), 195–196. (in Chinese).

[B19] Jia L. Jia Q. Yang J. Y. Jia R. Zhang H. (2018). Efficacy of probiotics supplementation on chronic kidney disease: a systematic review and meta-analysis. Kidney and blood Press. Res. 43 (5), 1623–1635. 10.1159/000494677 30380555

[B20] Jin Ke (2019). Observation on clinical effect of Jinkui Shenqi pills in treatment of spleen-kidney Yang deficiency type diabetic nephropathy. Clin. Pract. Integr. Chin. West. Med. 19 (04), 21–23.

[B21] Kelly T. N. Bazzano L. A. Ajami N. J. He H. Zhao J. Petrosino J. F. (2016). Gut microbiome associates with lifetime cardiovascular disease risk profile among bogalusa heart study participants. Circ. Res. 119 (8), 956–964. 10.1161/CIRCRESAHA.116.309219 27507222 PMC5045790

[B22] Kovatcheva-Datchary P. Nilsson A. Akrami R. Lee Y. S. De Vadder F. Arora T. (2015). Dietary fiber-induced improvement in glucose metabolism is associated with increased abundance of Prevotella. Cell Metab. 22 (6), 971–982. 10.1016/j.cmet.2015.10.001 26552345

[B23] Krishnan S. Alden N. Lee K. (2015). Pathways and functions of gut microbiota metabolism impacting host physiology. Curr. Opin. Biotechnol. 36, 137–145. 10.1016/j.copbio.2015.08.015 26340103 PMC4688195

[B24] Leustean A. M. Ciocoiu M. Sava A. Costea C. F. Floria M. Tarniceriu C. C. (2018). Implications of the intestinal microbiota in diagnosing the progression of diabetes and the presence of cardiovascular complications. J. Diabetes Res. 2018, 5205126. Published 2018 Nov 12. 10.1155/2018/5205126 30539026 PMC6260408

[B25] Li J. Zhao F. Wang Y. Chen J. Tao J. Tian G. (2017). Gut microbiota dysbiosis contributes to the development of hypertension. Microbiome 5 (1), 14. 10.1186/s40168-016-0222-x 28143587 PMC5286796

[B26] Liu R. F. Zhao H. Shi H. J. (2019). Research progress of probiotics and cardiovascular disease in type 2 diabetes mellitus. J. Med. Inf. 32 (18), 29–31.

[B27] Liu Z. W. (2014). Efficacy evaluation of Jinkui Shenqi pills in the treatment of diabetic nephropathy. Chin. J. Basic Med. Chin. Med. 20 (06), 821–822+831.

[B28] Mazruei Arani N. Emam-Djomeh Z. Tavakolipour H. Sharafati-Chaleshtori R. Soleimani A. Asemi Z. (2018). The effects of probiotic honey consumption on metabolic status in patients with diabetic nephropathy: a randomized, double-blind, controlled trial. Probiotics Antimicrob. proteins 11, 1195–1201. 10.1007/s12602-018-9468-x 30218286

[B29] Ni J. Hu Z. Wu X. (2013). Clinical observation on the adjuvant treatment of Penicillium batoides Cs-4 bacteria powder in diabetic nephropathy. Chin. J. Traditional Chin. Med. 31 (09), 1974–1976.

[B30] Oliveira R. B. Canuto L. P. Collares-Buzato C. B. (2019). Intestinal luminal content from high-fat-fed prediabetic mice changes epithelial barrier function *in vitro* . Life Sci. 216, 10–21. 10.1016/j.lfs.2018.11.012 30414427

[B31] Pedersen H. K. Gudmundsdottir V. Nielsen H. B. Hyotylainen T. Nielsen T. Jensen B. A. H. (2016). Human gut microbes impact host serum metabolome and insulin sensitivity. Nature 535 (7612), 376–381. 10.1038/nature18646 27409811

[B32] Qi S. Yin Z. Yan Z. (2020). Mechanism of yellow sunflower capsules on the intestinal microbiota in patients with diabetic nephropathy [J/OL]. Chin. J. Traditional Chin. Med. 1-8.

[B33] Qi Y. F. Ren L. F. Cao X. L. (2019). Intestinal flora composition and its relationship with sex and age in healthy people in Shanxi Province. Chin. J. Microecology 31 (10), 1117–1123.

[B34] Rizzatti G. Lopetuso L. R. Gibiino G. Binda C. Gasbarrini A. (2017). Proteobacteria: a common factor in human diseases. Biomed. Res. Int. 2017, 9351507. 10.1155/2017/9351507 29230419 PMC5688358

[B35] Ruiz-Ortega M. Rodrigues-Diez R. R. Lavoz C. Rayego-Mateos S. (2020). Special Issue"Diabetic nephropathy: diagnosis, prevention and treatment. J. Clin. Med. 9 (3), 813. 10.3390/jcm9030813 32192024 PMC7141346

[B36] Salguero M. V. Al-Obaide M. a. I. Singh R. Siepmann T. Vasylyeva T. L. (2019). Dysbiosis of Gram-negative gut microbiota and the associated serum lipopolysaccharide exacerbates inflammation in type 2 diabetic patients with chronic kidney disease. Exp. Ther. Med. 18 (5), 3461–3469. 10.3892/etm.2019.7943 31602221 PMC6777309

[B37] Shimizu M. Hashiguchi M. Shiga T. Tamura H. o. Mochizuki M. (2015). Meta-analysis: effects of probiotic supplementation on lipid profiles in normal to mildly hypercholesterolemic individuals. PLoS One 10 (10), e0139795. Published 2015 Oct 16. 10.1371/journal.pone.0139795 26473340 PMC4608827

[B38] Su J. Chen H. (2020). Observation on the efficacy of bifidobacteria triple live bacteria combined with Candesartan in the treatment of diabetic nephropathy. Chin. Med. Innov. 17 (19), 1–4.

[B39] Sun H. Saeedi P. Karuranga S. Pinkepank M. Ogurtsova K. Duncan B. B. (2022). IDF Diabetes Atlas: global, regional and country-level diabetes prevalence estimates for 2021 and projections for 2045. Diabetes Res. Clin. Pract. 183, 109119. 10.1016/j.diabres.2021.109119 34879977 PMC11057359

[B40] Tan F. Zhou Z. (2021). Bifidobacteria and intestinal diseases and diabetes. J. Difficult Dis. 20 (02), 194–198.

[B41] Tan J. Mckenzie C. Potamitis M. Thorburn A. N. Mackay C. R. Macia L. (2014). The role of short-chain fatty acids in health and disease. Adv. Immunol. 121, 91–119. 10.1016/B978-0-12-800100-4.00003-9 24388214

[B42] Tan S. H. Candasamy M. Bhattamisra S. K. (2019). Diabetic nephropathy: an update on pathogenesis and drug development. Diabetes Metab. Syndr. 13 (1), 754–762. 10.1016/j.dsx.2018.11.054 30641802

[B43] Tett A. Pasolli E. Masetti G. Ercolini D. Segata N. (2021). Prevotella diversity, niches and interactions with the human host. Nat. Rev. Microbiol. 19 (9), 585–599. 10.1038/s41579-021-00559-y 34050328 PMC11290707

[B44] Tian X. Yong Z. Huachuan Z. X. (2020). Application of zhong jingfang in diabetic nephropathy. J. Pract. Chin. Med. Intern. Med. 34 (11), 4.

[B45] Wang Q. Yang Y. (2019). Research progress of intestinal flora disturbance on diabetes and diabetic nephropathy. Med. Rev. 25 (03), 530–534. (in Chinese).

[B46] Wang X. F. Chen W. Huang G. S. (2021). Study on the mechanism of action of Jinkui Shenqi pills in treating diabetic nephropathy based on network pharmacology. J. Pract. Chin. Med. Intern. Med. 35 (12), 24–27+153-157.

[B47] Waters J. L. Ley R. E. (2019). The human gut bacteria Christensenellaceae are widespread, heritable, and associated with health. BMC Biol. 17 (1), 83. 10.1186/s12915-019-0699-4 31660948 PMC6819567

[B48] Xiaoyu Z. (2002). Guiding Principles of clinical research on new Chinese medicine. Guid. Princ. Clin. Res. New Chin. Med.

[B49] Yuan Y. Nie S. (2020). Effect of Jinkui Shenqi Pills on blood glucose and renal function in patients with yin-yang deficiency type diabetic nephropathy. Mod. Chin. Med. 40 (05), 70–72+76.

[B50] Zafar H. Saier M. H. Jr (2021). Gut Bacteroides species in health and disease. Gut Microbes 13 (1), 1–20. 10.1080/19490976.2020.1848158 PMC7872030 33535896

[B51] Zaky A. Glastras S. J. Wong M. Y. W. Pollock C. A. Saad S. (2021). The role of the gut microbiome in diabetes and obesity-related kidney disease. Int. J. Mol. Sci. 22 (17), 9641. Published 2021 Sep 6. 10.3390/ijms22179641 34502562 PMC8431784

[B52] Zhao L. Xie Q. Etareri E. S. Liu D. Dong J. Ping L. (2021). Bifidobacterium dentium N8 with potential probiotic characteristics prevents LPS-induced intestinal barrier injury by alleviating the inflammatory response and regulating the tight junction in Caco-2 cell monolayers. Food Funct. 12 (16), 7171–7184. 10.1039/d1fo01164b 34269367

[B53] Zhu Na (2018). Meta-analysis of efficacy and safety of microecological preparations in maintaining dialysis patients. Nanchang Nanchang Univ.

