# Corrigendum: Effects of *Bifidobacterium bifidum* tetragonum tablets and Jin Gui Ren Qi Pill on intestinal flora and metabolism in patients with diabetic kidney disease

**DOI:** 10.3389/fphar.2024.1480859

**Published:** 2024-09-12

**Authors:** Cheng-Yu Zhang, Dong-jie Yue, Di Wang, Fei-fei Wu

**Affiliations:** ^1^ Department of Endocrinology and Metabolism, The Fifth People’s Hospital of Chongqing, Chongqing, China; ^2^ Heping Hospital Affifiliated to Changzhi Medical College, Changzhi, Shanxi, China; ^3^ Zhengzhou Second People’s Hospital, Zhengzhou, Henan, China; ^4^ Department of Endocrinology and Metabolism, Binzhou People’s Hospital, Binzhou, Shandong, China; ^5^ Department of Endocrinology and Metabolism, Heping Hospital Affiliated to Changzhi Medical College, Changzhi, Shanxi, China

**Keywords:** diabetic kidney disease, Bifidobacterium bifidum tetragonum tablets, Jin Gui Ren Qi Pill, intestinal flor, serum total cholesterol

In the published article, there was an error regarding the affiliations for Cheng-Yu Zhang. As well as having **Affiliation** 1, they should also have Heping Hospital Affiliated to Changzhi Medical College, Changzhi, China.

In the published article, there was an error regarding the affiliations for Dong-jie Yue. As well as having **Affiliation** 3, they should also have Heping Hospital Affiliated to Changzhi Medical College, Changzhi, China.

In the published article, there was an error regarding the affiliations for Di Wang. As well as having **Affiliation** 4, they should also have Heping Hospital Affiliated to Changzhi Medical College, Changzhi, China.

The authors apologize for these errors and state that this does not change the scientific conclusions of the article in any way. The original article has been updated.

